# Universal and individual characteristics of postural sway during quiet standing in healthy young adults

**DOI:** 10.14814/phy2.12329

**Published:** 2015-03-16

**Authors:** Tomohisa Yamamoto, Charles E Smith, Yasuyuki Suzuki, Ken Kiyono, Takao Tanahashi, Saburo Sakoda, Pietro Morasso, Taishin Nomura

**Affiliations:** 1 Graduate School of Engineering Science, Osaka UniversityToyonaka, Japan; 2 Department of Statistics, North Carolina State UniversityRaleigh, North Carolina, USA; 3 Department of Neurology, Osaka University Graduate School of MedicineOsaka, Japan; 4 Department of Neurology, Toneyama National HospitalOsaka, Japan; 5 RBCS Department, Fondazione Istituto Italiano di TecnologiaGenoa, Italy

**Keywords:** Intermittent control, postural control, postural sway, slow component

## Abstract

The time course of the center of pressure (CoP) during human quiet standing, corresponding to body sway, is a stochastic process, influenced by a variety of features of the underlying neuro-musculo-skeletal system, such as postural stability and flexibility. Due to complexity of the process, sway patterns have been characterized in an empirical way by a number of indices, such as sway size and mean sway velocity. Here, we describe a statistical approach with the aim of estimating “universal” indices, namely parameters that are independent of individual body characteristics and thus are not “hidden” by the presence of individual, daily, and circadian variations of sway; in this manner it is possible to characterize the common aspects of sway dynamics across healthy young adults, in the assumption that they might reflect underlying neural control during quiet standing. Such universal indices are identified by analyzing intra and inter-subject variability of various indices, after sorting out individual-specific indices that contribute to individual discriminations. It is shown that the universal indices characterize mainly slow components of sway, such as scaling exponents of power-law behavior at a low-frequency regime. On the other hand, most of the individual-specific indices contributing to the individual discriminations exhibit significant correlation with body parameters, and they can be associated with fast oscillatory components of sway. These results are consistent with a mechanistic hypothesis claiming that the slow and the fast components of sway are associated, respectively, with neural control and biomechanics, supporting our assumption that the universal characteristics of postural sway might represent neural control strategies during quiet standing.

## Introduction

Postural sway during human quiet standing is often quantified by measuring the motion of the Center of Pressure (CoP), namely the point of application of the ground reaction force vector. CoP shift profiles are closely related to the sway of the Center of Mass (CoM) during quiet standing (Morasso et al. 1999). Thus, motion of the standing body can be estimated from CoP patterns with an acceptable accuracy either in the context of the single-inverted pendulum model (Morasso et al. 1999; Lafond et al. 2004) or the double pendulum model with hip and ankle joints (Colobert et al. 2006). Characterizing CoP motion is of critical importance for understanding neural mechanisms of postural control (e.g., Winter et al. 1998; Peterka 2002; Bottaro et al. 2005; Kiemel et al. 2006; Kim et al. 2008) as well as for better diagnosing severity of neurological diseases with postural instability (e.g., Horak et al. 1992; Rocchi et al. 2002; Maurer et al. 2003; Visser et al. 2008).

CoP complex fluctuations can be modeled as a two-dimensional stochastic process (Carroll and Freedman 1993; Collins and De Luca 1994; Loughlin et al. 1996), in the anterior-posterior (AP) and medio-lateral (ML) directions on the horizontal plane. Due to complexity, CoP time-series have been characterized by a number of simple, usually scalar valued measures or indices (Collins and De Luca 1993; Prieto et al. 1996; Baratto et al. 2002; Jacono et al. 2004; van der Kooij et al. 2011), such as sway size (Seigle et al. 2009), mean sway velocity (Raymakers et al. 2005), and scaling exponents (Collins and De Luca 1994; Milton et al. 2009). Since each index can measure only a limited aspect of the process, two sway time-series characterized by the same sway size, for example, can be accompanied by completely different temporal patterns. A set of indices that capture different (i.e., uncorrelated and/or independent) aspects of sway might be able to describe details of the process (Prieto et al. 1996). Alternatively, some aspect of sway characterized by an appropriate index might be able to reflect inherent neural control of postural dynamics, and others might represent merely individual motor habits and/or body-parameter-dependent biomechanics (Chiari et al. 2002; Hue et al. 2007).

The current study was motivated by our preliminary work that examined differences in CoP signals among healthy young, healthy elderly and elderly patients with Parkinson's disease (Yamamoto et al. 2011), showing that the population of patients exhibited a postural sway of significantly smaller size than the healthy population. This fact seemed paradoxical and inconsistent with the symptom of postural instability, which is typical in most patients with Parkinson's disease, if we take into account that large sway size is often considered implicitly as a typical sign of postural instability (Frenklach et al. 2009). However, this apparent paradox might be consistent with the postural inflexibility observed by Horak et al. (1992). On the other hand, as noted by Rocchi et al. (2002), there is no agreement among researchers on the specific features of sway in quiet standing that characterize Parkinson's disease, although the term “abnormal” is frequently used to describe such patterns, with the implicit assumption that abnormal sway means excessive sway. In any case, the issue of physiological versus pathological sway size should be carefully reexamined in the context of optimal motor variability (Stergiou et al. 2006).

Body sway during quiet standing in healthy subjects shows subject-dependent variability. In other words, CoP signals might exhibit less trial-to-trial variability within individuals. Instead, it might be subject specific over a sequence of multiple measurements at different occasions for a given individual. Indeed, Santos et al. (2008) showed that CoP signals exhibit less daily and circadian variability than intuitively expected, and thus are more reliable within individuals. Moreover, CoP can be used for systematic individual discriminations (Demura et al. 2001). For example, it has been shown that CoP velocity is strongly correlated with body weight, where the amount of weight-dependent individual differences is comparable with the standard deviation of CoP velocity across subjects (Teasdale et al. 2007). Individual specificity and reliability of CoP signals imply that comparisons in the values of an index between populations of subjects without taking into account the individual specificity might possibly lead to inappropriate interpretations of postural functions such as stability and flexibility (van der Kooij et al. 2011), which could also be the case in the above-mentioned clinical situations.

This study aims to elucidate universal indices that can measure postural characteristics common across healthy young subjects, independent of body parameters and not hidden by the presence of individual, daily, and circadian variations of sway. Such universal indices, if any, might reflect the origins of postural fluctuation, i.e., inherent neural control mechanisms that induce postural sway. Several theories have been formulated about such mechanisms. In particular, we may expect that indices associated with the slow components of sway in the 0.1–0.5 Hz frequency range, either nonoscillatory (Kiemel et al. 2006) or oscillatory (Nomura et al. 2013), might be highlighted as major factors of universal indices, since the slow components have been shown to account for the majority of sway variance during quiet stance (Kiemel et al. 2002) and they are considered as key elements for understanding neural control of upright standing (Kiemel et al. 2006). However, there is still active debate about the origin of such slow components: (1) are they the manifestation of imperfect estimation with (Loram et al. 2005, 2011) or without (Kiemel et al. 2006) intermittency in the control loop? (2) are they due to a slowly migrating reference point defined by a central command (Zatsiorsky and Duarte 1999)? (3) are they due to the intermittency in delay feedback control (Insperger 2006; Bottaro et al. 2008; Asai et al. 2009; Milton et al. 2009; Suzuki et al. 2012)? In any case, there is agreement that slow postural dynamics is determined by the neural control, not by unspecified colored, long-term correlated noise perturbing the upright stance. There is also agreement about the mechanistic origin of the fast oscillatory components, in the 0.5–1.0 Hz frequency range, and the mechanical dynamics of single- and double-inverted pendulum-like body (Kiemel et al. 2002; Creath et al. 2005), for the faster components in the 1.0–2.5 Hz frequency range.

The statistical analysis, adopted in this study for elucidating universal indices, is model-free (hypothesis-free) and thus it might be unable to strongly suggest which hypothesis is most physiologically plausible. However, we are confident that it could be beneficial for understanding neural control of upright posture provided that reasonable correspondences can be found between sets of universal indices and model-based functional mechanisms for stabilizing upright posture. This is because a process of finding universal indices is completely independent of hypothetical postural control mechanisms that have been proposed previously, and coincidental matches of universal indices with some of the hypothetical control mechanisms might imply that aspects of postural sway characterized by the universal indices are associated with those control mechanisms.

In the Methods section, an experimental protocol for measuring CoP signals during quiet standing is described first. Then, a number of sway indices for characterizing such signals are introduced. Criteria for evaluating universality and individual specificity for those indices are defined, based on which, the indices are classified into three groups: (1) universal group, (2) individual-specific group, (3) mixed group. Statistical methods used for performing, improving and validating the classification are described. The Results section summarizes our classification. We then discuss about how the statistical index classification can be interpreted.

## Methods

### Experimental methods

Measurements of CoP signals were performed with sixteen healthy young adult men during quiet standing on a force platform (Model OR-6-5-1000, AMTI, Watertown, MA) with eyes open. Each subject was instructed to place his bare feet along a *V*-shaped guide marked on the platform, in such a way that the ankles were aligned with the medio-lateral (ML) axis of the platform and the two malleoli were equally separated about 1 cm from the anterior-posterior (AP) axis of the platform: thus, the platform origin coincided roughly to the center of gravity (vertical projection of CoM) of the ideal vertically upright body, in order to enable intertrials and intersubject comparison of CoP position with respect to the ankle position (the origin of the force plate). Subjects were instructed to keep their gaze at a fixation point displayed at eye-level height about 2 m away from them. Measurements were performed at five different times of a day (10:00 am, 12:00 pm, 2:00 pm, 4:00 pm, 6:00 pm) for three contiguous days. For each measurement session, four trials were performed of 70 s quiet standing. Thus, in the 3 days, 60 CoP time series were acquired from each subject. Since one subject (Subject-15) reported a consistent lack of sleep, the sway data from this subject were eliminated from the analysis. Thus, the data used for the following analysis were from 15 subjects, with a total of 900 sway data. Means and standard deviations characterizing those subjects were as follows; age: 23.4 ± 1.8 years old, height: 171.0 ± 5.4 cm, weight: 66.3 ± 9.5 kg. All subjects provided written informed consent to participate in this research, which has been approved by the ethical committee for human studies at Graduate School of Engineering Science, Osaka University.

Both components of the CoP data were digitized, namely the component in the ML-direction (CoP-ML) and that in the AP-direction (CoP-AP), using a 16-bit A/D converter, with a sampling frequency of 60 Hz; then they were low-pass filtered off-line, using a fourth-order zero-phase-lag Butterworth filter with a cut-off frequency of 10 Hz, before the analysis described below.

### Sway indices

From the stored CoP data, a large number of sway indices – 73 – were computed: most of them were already proposed in the literature and some of them were introduced in this study. The complete list is shown in Table1, which reports the index numbers, names, and brief descriptions. See the Supporting Information for detailed definitions of the indices. A preliminary normalization of the data was carried out by detecting indices that were not characterized by a normal distribution over the whole population of subjects and the whole set of trials. In such cases, a logarithmic transformation was carried out in the assumption that such indices values might exhibit a log-normal distribution. The following step was to standardize each index in such a way to exhibit a null mean and a unitary standard deviation over the whole set of trials for all the subjects. In particular, if we denote with 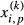 the original *k*-th index (*k* ∈ {1,···,73}), the subscripts *i* runs through *i *= 1, ···, *N* with *N *=* *900 being the number of total trials for counting trials across all subjects, and through *i* = 1, ···, *n* with *n* = 60 being the number of individual total trials for counting trials within each individual. The subscript *p* ∈ {1, ···, 15} represents the subject number. The corresponding standardized *k*-th index or *z*-scores were computed as follows:

**Table 1:** The list of 73 indices for characterizing CoP time-series. Indices with “^*^” were defined for both CoP-ML and CoP-AP. Indices of their numbers with and without parentheses represent that they were obtained for CoP-AP and CoP-ML, respectively. Indices with “^*^^*^” were defined for CoP of planar movement, CoP-ML and CoP-AP, where indices without parentheses are for planar movement, and those with parentheses are for CoP-ML and CoP-AP, in this order. Indices with italic numbers did not pass the normality test, thus they exhibited non-Gaussian distributions

Index no.	Index name	Description	References
1(2)	Mean^*^	Mean position of sway	Vuillerme et al. (2002), Kirby et al. (1987)
3	log-Area	Log of 95% confidence ellipse area	Rocchi et al. (2002), Maurer et al. (2003), Schieppati et al. (1994))
4	log-Axis1	Log of the size of major axis of 95% confidence ellipse	Agostini et al. (2013)
5	log-Axis2	Log of the size of minor axis of 95% confidence ellipse	Agostini et al. (2013)
*6*	Angle	Absolute value of angle between major axis and ML axis	Rocchi et al. (2002)
7(8)	Mean-cross^*^	The number of mean CoP crosses	
9(12)	Slope-L^*^	Slope at low-frequency band in PSD of CoP	Yamamoto et al. (2011), Asai et al. (2009), van der Kooij et al. (2005)
10(13)	Slope-H^*^	Slope at high-frequency band in PSD of CoP	Yamamoto et al. (2011), Asai et al. (2009), van der Kooij et al. (2005)
11(14)	Critical-freq^*^	Critical frequency at which two regression lines of PSD of CoP intersect	
15(16)	Zero-cross-V^*^	The number of zero crosses of low-pass filtered CoP velocity	
17	log-LNG	Log of total path length of CoP trajectory	Chastan et al. (2008), Stylianou et al. (2011)
18	log-LNG/Area	Log of total path length of CoP trajectory divided by 95% confidence ellipse area	Demura et al. (2001)
19(21)	log-Alpha^*^	Log of shape parameter of Gamma distribution fitted to the duration of mean CoP velocity crosses	
20(22)	Beta^*^	Scale parameter of Gamma distribution fitted to the duration of mean CoP velocity crosses	
23	MT3	Mean time interval between successive peaks on Sway-Density Curve at *R *=* *3	Jacono et al. (2004)
24	MP3	Mean peak value on Sway-Density Curve at *R *=* *3	Jacono et al. (2004)
25	MD3	Mean distance in AP-ML plane between successive peaks on Sway-Density Curve at *R *=* *3	Jacono et al. (2004), Popa et al. (2007)
26	Mean-MT	Mean MT value for *R *∈* *[2, 5]	Jacono et al. (2004)
27	log-Slope-MP	Log of slope of regression line of graph for MP versus *R *∈* *[2, 5]	Jacono et al. (2004)
28	Mean-MD	Mean MD value for *R *∈* *[2, 5]	Jacono et al. (2004), Popa et al. (2007)
29	FD	Fractal dimension	Prieto et al. (1996)
30	log-Area-SW	Log of mean triangle area enclosed by mean CoP position and two consecutive points	Prieto et al. (1996), Agostini et al. (2013)
31(32,33)	MFREQ^*^^*^	Mean frequency of a circular motion with a radius equal to mean amplitude	Prieto et al. (1996)
34(37,40)	log-Power^*^^*^	Log of total power of CoP	Prieto et al. (1996)
35(*38*,41)	PF50^*^^*^	50% power frequency of CoP	Prieto et al. (1996)
36(39,42)	PF95^*^^*^	95% power frequency of CoP	Prieto et al. (1996), Rocchi et al. (2002)
43(*47*,51)	D-short^*^^*^	Diffusion coefficient of CoP at short-term region	Collins and De Luca (1993)
*44*(*48*,*52*)	D-long^*^^*^	Diffusion coefficient of CoP at long-term region	Collins and De Luca (1993)
*45*(*49*,*53*)	Critical-Δt-linear^*^^*^	Time interval at the intersection of two regression lines on linear-scale stabilogram diffusion plot	Collins and De Luca (1993), Maurer et al. (2004)
*46*(*50*,*54*)	Critical-D-linear^*^^*^	Mean square value at Critical-Δt-linear on linear-scale stabilogram diffusion plot	Collins and De Luca (1993), Maurer et al. (2004)
55(59,63)	Slope-short^*^^*^	Slope of stabilogram at short-term region on log-scale stabilogram diffusion plot	Collins and De Luca (1993), Maurer et al. (2004)
56(60,64)	Slope-long^*^^*^	Slope of stabilogram at long-term region on log-scale stabilogram diffusion plot	Collins and De Luca (1993), Maurer et al. (2004)
57(61,65)	Critical-Δt-log^*^^*^	Time interval at the intersection of two regression lines on log-scale stabilogram diffusion plot	Collins and De Luca (1993), Maurer et al. (2004), Maurer and Peterka (2005)
58(62,66)	Critical-D-log^*^^*^	Mean square value at Critical-Δt-log on log-scale stabilogram diffusion plot	Collins and De Luca (1993), Maurer et al. (2004), Maurer and Peterka (2005)
67(68,69)	log-RMS^*^^*^	Log of root mean square distance of CoP	Rocchi et al. (2002), Maurer et al. (2004), Agostini et al. (2013)
70(71,72)	log-MV^*^^*^	Log of mean CoP velocity	Prieto et al. (1996), Kouzaki and Masani (2012), Agostini et al. (2013)
73	Flattening	Flattening of 95% confidence ellipse	Agostini et al. (2013)



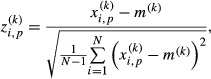
1

where

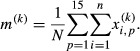
2

All statistical analyses were carried out using either MATLAB Statistical toolbox (The Mathworks, Natick, MA) or SAS (SAS Institute, Cary, NC).

### Classification of indices

**Figure 1:**
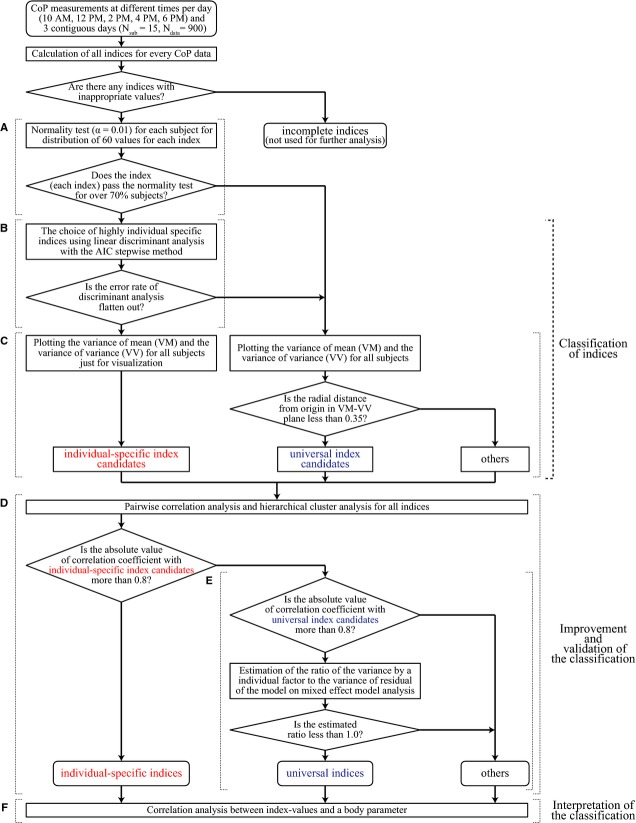
Flow chart for the classification of indices. It was composed of three steps; namely, classification, improvement and validation, and interpretation of the classification. See Methods for details.

As explained in the previous subsection, after normalization and standardization of the 73 indices, namely for the set of 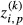, the next step would be to divide them into 3 classes: (1) individual-specific indices, (2) universal indices, (3) other indices. The procedure is shown in Fig.1. For each index, each trial, and each subject, we computed individual means and variances. The idea, detailed in Fig.1 is that an index is a candidate for the universal class if its means and variances are common across subjects. Contrastingly, if the individual means of a given index vary significantly across subjects and, moreover, if the individual variance is small, the index might represent an individual-specific feature of sway. For classifying the indices, we first determined possible candidates of individual-specific indices using linear discriminant analysis, and they were sorted out from the overall set before selecting candidates of universal indices.

### Normality test

The linear discriminant analysis for selecting candidates of individual-specific indices requires normal distribution for every set of 60 values of each index for each subject. Thus, the Lilliefors' normality test (*P* < 0.01) was applied to each of 73 sets of indices for each subject. An index was included in the linear discriminant analysis, if it passed the normality test for more than 70% of the subjects (11 or more out of 15 subjects). This step corresponds to Fig.1A based on which *M* indices out of 73 indices were used for the linear discriminant analysis.

### Linear discriminant analysis

In the linear discriminant analysis for selecting candidates of individual-specific indices, linear discriminant functions were configured so that they could discriminate every individual correctly as much as possible from 15 subjects. Since it was expected that not all but only a limited number of indices are useful for the discrimination, optimally selected *q* indices from *M* normally distributed indices were used for the analysis. In the analysis, *N*(=*n* × *p* = 900) points (index vectors) distributed in the *q*-dimensional index space were considered. The linear discriminant function that could discriminate subject-*p* from subject-*p*′ forms a (*q*−1)-dimensional hyperplane, and it was determined so that it could separate 60 index vectors for subject-*p* from those for subject-*p*′. The “optimal” set of *q* indices used for the individual discrimination was determined using a stepwise selection technique based on the Akaike Information Criterion (AIC) as described below, since some indices might highly contribute to discriminating each individual, some might be redundant depending on the combination of indices due to correlations among indices, or some do not contribute to the discrimination at all. The *q* indices selected in this way out of *M* normally distributed indices were considered as candidates of individual-specific indices. This step corresponds to Fig.1B. See Appendix A1 for details of how the linear classifier for the individual discrimination that utilizes *q* indices was obtained.

### Selection of the set of indices using AIC

Selections of the optimal *q* indices for the discrimination were performed using the AIC-based stepwise method (Fujikoshi 1985), in which the optimal combination of indices that minimizes the AIC was identified. Note that, in general, the larger the number of indices used, the more correct will be the discrimination, although a too large number may cause an over-fitting problem, which can be avoided by the use of AIC. The iterative algorithm is articulated in Appendix A2.

### Determination and validation of individual-specific indices

The iterative algorithm described above provides a list of indices, ordered according to a decreasing degree of contribution to the individual discrimination. Candidates of individual-specific indices were determined based on the apparent error rate and the error rate evaluated in the cross-validation procedure (leave-one-out method). In the latter, the error rate was calculated by examining the discriminant performance for each of 15 subjects using the optimal linear classifier that uses each of *q* indices (*q *= 1, 2, ···, *M*) in the iterative algorithm. Candidates of individual-specific indices were determined as the set of indices for which the apparent error rate and the error rate were converged to a small value below about 10%.

### Universality and individual-specificity of sway indices

For each subject, 60 values were calculated for each index, thus providing individual mean and variance of the index for each subject. Candidates of the universal indices were determined by examining variability of the individual means (VM: Variance of individual Means) and the individual variances (VV: Variance of individual Variances) across subjects for each index. In this way, the candidates of individual-specific indices obtained by the linear discriminant analysis could be validated by using a VM-VV plot for each index. Namely, for the individual-specific indices, we expect the subject-to-subject variability of individual means to be large but the variability of individual variances to be small, and thus they should be plotted far from the origin along the VM-axis of the VM-VV plane.

On the other hand, for universal indices, we expect the subject-to-subject variability of individual means and individual variances to be both small, and thus they should be plotted close to the origin of the VM-VV plane. In this study, if a distance between a VM-VV point of an index and the origin in the VM-VV plane was less than or equal to 0.35, that index was considered as a universal index candidate. This step corresponded to Fig.1C.

Finally, for the third class of indices, the subject-to-subject variability of individual means should have intermediate values and they should appear in a different area of the VM-VV plane, away from the origin and the VM-axis.

### Pairwise correlation and cluster analysis of indices

In order to improve the classification of individual-specific and universal index candidates, a pairwise correlation analysis among indices was performed. More specifically, correlation coefficients between all possible combinations of two indices were calculated. If a correlation coefficient with one of the individual-specific index candidates was greater than 0.8 for an index that had not been selected as an individual-specific index candidates in the previous procedure, that index was shifted into the group of individual-specific indices. This step corresponds to Fig.1D, by which the determination of individual-specific indices was completed.

Similarly, if a correlation coefficient with one of the universal index candidates was greater than 0.8 for an index that had not been selected as a universal index candidate in the previous procedure, that index was shifted into the group of universal indices. This step corresponded to Fig.1E. However, as shown later in the result section, this rule did not apply to the experimental data.

A hierarchical cluster analysis with group average method was then performed to classify indices into several clusters that were correlated with each other. This analysis could be a validation of the classification by the linear discriminant analysis, and also useful for general studies of postural control to clarify sets of indices that involve similar (or redundant) information about CoP time-series. In the cluster analysis, a distance between two indices was defined using the correlation coefficient between those indices. More specifically, for a correlation coefficient *r* between two indices, the distance between those indices was defined as 1−*r*. Distances between all possible pairs of indices in two clusters were calculated, and the average value of those distances was considered as the distance between the two clusters.

### Linear mixed-effect model analysis

Finally, a linear mixed-effect model analysis was performed for the obtained universal index candidates to further improve the determination of universal indices. The linear mixed-effect model analysis is useful to clarify factors of variance of those indices. The global variance *V* of an index is indeed a combination of the variances attributed, respectively, to the subjects *σ*_sub_, to the days *σ*_day_, to the times *σ*_time_, to the interactions among these three factors *σ*_sub × day_, *σ*_sub × time_, *σ*_day × time_, and to a generic variance *σ*_e_:

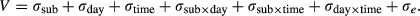
3

If, for a candidate of the universal index group, the amount of variance attributed to the subject is smaller than the error variance, it is plausible to conclude that variability of the index is not due to subjects but just to a natural variability, thus confirming that the index was indeed universal. More specifically, the ratio *σ*_sub_/*σ*_e_ was calculated for each candidate of universal index group, and if the ratio was less than or equal to 1.0 for an index, the index was confirmed as universal.

### Correlation between body parameters and sway indices

In order to gain insights about physiological and/or biomechanical meanings from the index-classification and to examine whether the universality and the individual specificity of indices reflect biomechanical features of the body, a correlation analysis between body parameters and values of each index was performed. It was expected that universal indices were not correlated significantly with body parameters since universal indices represent common features of CoP across subjects, whereas it would be the case for individual-specific indices.

More specifically, correlation coefficients between the moment of inertia for each of 15 subjects and values of each index were calculated. This step corresponded to Fig.1F. The moment of inertia for each subject was calculated by *mh*^2^ where *m* and *h* were the mass and height of the subject. Values of the inertia were standardized as zero mean and unit variance across subjects, respectively, prior to the correlation analysis.

## Results

### Measurements of CoP patterns

**Figure 2:**
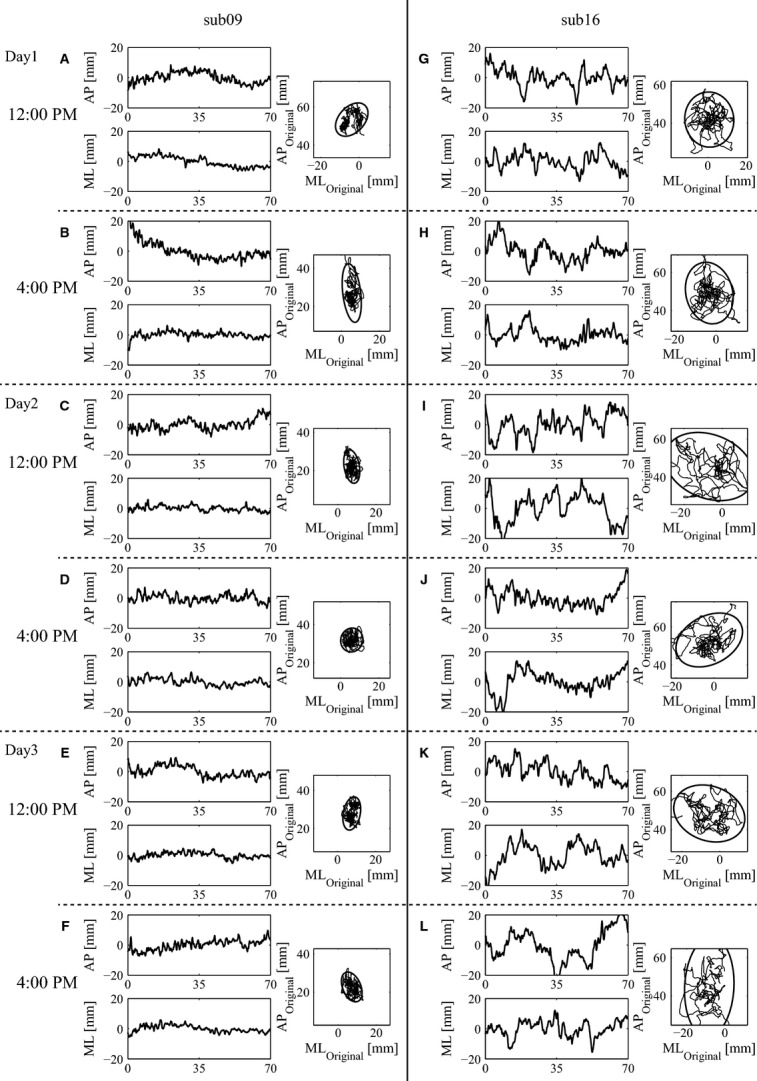
Examples of CoP patterns (planar CoP trajectory, CoP-AP and CoP-ML) for two different subjects measured at different circadian times and different days. (A)–(F): CoP data from subject-09. (G)–(L): CoP data from subject-16. For each subject, from the top to the bottom panels, the measurements were performed at 12:00 pm of Day 1, 4:00 pm of Day 1, 12:00 pm of Day 2, 4:00 pm of Day 2, 12:00 pm of Day 3, and 4:00 pm of Day 3.

Figure2 exemplifies CoP trajectories traced in the support plane, and CoP-AP and CoP-ML time-series for two different subjects, measured at different circadian times of three contiguous days. In this particular case, the intersubject differences are quite apparent and clearly greater than the day-dependent intra-subject variability. However, aspects commonly shared by the two subjects are not obvious. Clarification of such universal features of sway, if any, require the quantitative characterizations of the sway data presented in the methods and described in the following.

### Values of the indices

Values of the 73 indices were evaluated for each of the 900 CoP time-series from all subjects. Eight indices exhibited apparently inappropriate values or failure in obtaining index-values for certain sets of data. Those indices were associated with two specific features of the sway patterns: (1) log-log plots of the power spectral density functions of the posturographic data; (2) log-scaled stabilogram diffusion plots. In the former case, two indices (11 and 14) characterize the critical frequency that separates low- and high-frequency bands. In the latter case, six indices (57, 58, 61, 62, 65, and 66) are related to the critical time-lag that separates short- and long-term scaling regimes. The problem is that the algorithm used for estimating those indices assume a double power law behavior, which is indeed found in most of the subjects. However, in some case, the data are characterized by a single-power-law-like behavior and then the algorithm fails.

In this way, eight indices (numbered 11, 14, 57, 58, 61, 62, 65 and 66 in Table1) were eliminated and thus, the number of indices used for the following classification was reduced to 65.

### Normality test of the indices for each subject

**Figure 3:**
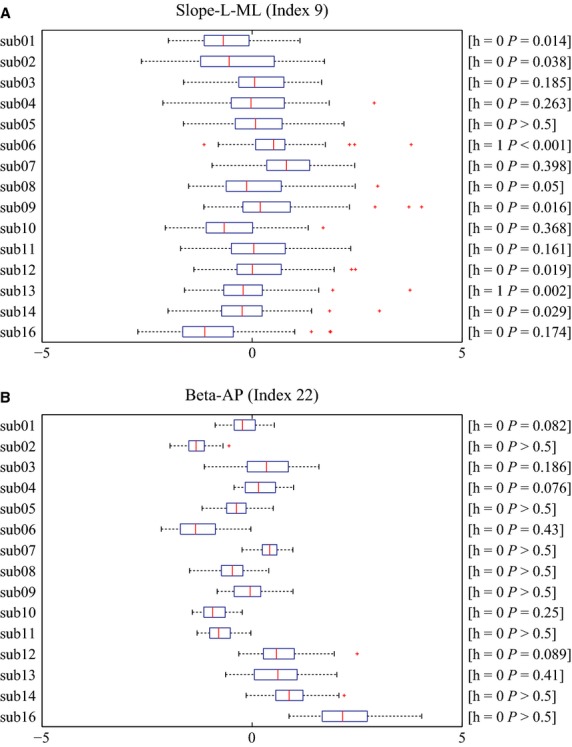
Box-plot of every subject for (A) Slope-L-ML (Index 9) and (B) Beta-AP (Index 22). (A) Slope-L-ML (Index 9) was normally distributed in most of the subjects. *h* and *P*-values for each panel represent the results of Lilliefors' test examining the null hypothesis that the data comes from a normal distribution. *h* = 1 if the test rejects the null hypothesis at 1% significance level, and *h* = 0 otherwise. The individual means of Slope-L-ML index for all subjects were close to each other, which made the individual variances relatively large. (B) Beta-AP (Index 22) was also normally distributed in all subjects. The individual means of Beta-AP index were largely subject-dependent, which made the individual variances relatively small.

The Lilliefors' normality test was performed for each of the 65 remaining indices from each subject: 53 of them passed the test. Figure3 exemplifies individual box-plots of normally distributed index-values for two indices, namely the index 9 (Slope-L-ML: the slope of power law behavior, i.e., the scaling exponent in the log-log power spectral density at a low-frequency band for CoP-ML) and the index 22 (Beta-AP: the scale parameter of Gamma distribution that approximates the distribution of time intervals between changes in the sign of CoP-AP sway velocity). It is quite evident that the boxes in the former case were located almost centrally with similar mean and standard deviation values across subjects, whereas in the latter case the boxes exhibited varied means and standard deviations. This means that the index 9 is universal and the index 22 is individual.

Twelve indices that did not pass the normality test, namely indices 6, 38, 44, 45, 46, 47, 48, 49, 50, 52, 53 and 54. In particular, index 6 represents the absolute value of the angle between ML axis and major axis of confidence ellipse of CoP; indices 44, 48, and 52 represent the diffusion coefficients of CoP at long-term region obtained from the linearly-scaled stabilogram diffusion plot; indices 45, 49, and 53 represent the critical time-lag obtained from the linearly-scaled stabilogram diffusion plot; indices 46, 50, and 54 represent the mean square value of the above-mentioned indices 45, 49, and 53. In general, this means that the indices associated with the stabilogram diffusion plot mostly exhibited non-Gaussian distributions, which suggests necessity of careful use of those indices, despite their popularity in the recent studies (e.g., Doyle et al. 2008; T Hsiao-Wecksler et al. 2003; Bosek et al. 2005). Those indices were not used in the following linear discriminant analysis, i.e., they were eliminated from the candidates of individual-specific indices.

### Linear discriminant analysis for selecting candidates of individual-specific indices

**Figure 4:**
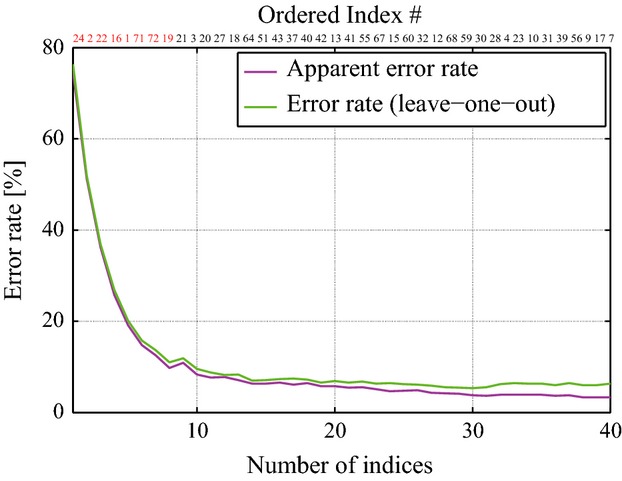
Apparent error rate and error rate of leave-one-out cross-validation as the function of number of indices used for the linear discriminant analysis. The order of indices was determined by the AIC-based stepwise method, where the indices were included into the linear classifier according to the order of indices. Red-color numbers represent the indices that were selected as the individual-specific index candidates.

The AIC-based stepwise method for determining the linear discriminant functions was applied for the selection of an optimal set of indices among the 53 normally distributed indices: 40 indices exhibited individual-discrimination ability, whereas the remaining 13 indices did not and were excluded for the selection of the optimal set. The selection criterion was based on the apparent error rate and the error rate in the cross-validation (leave-one-out method), which examined the rates such that the optimal linear discriminant functions failed in discriminating each individual. The 40 indices were ranked according to such error measures and were ordered from the one that contributed most to discriminating individuals to the one that contributed less. Figure4 plots the error measures of the ordered set of 40 indices (the actual index numbers are reported on top of the figure box). Of course both plots fall down monotonously and we chose, as a threshold, an error rate of 10%. This threshold identifies the following set of 8 indices as the best candidates of the group of individual-specific indices: 24, 2, 22, 16, 1, 71, 72, and 19 in a descending order of discriminating ability. The numbers of such indices are colored in red in the upper part of Fig.4. Moreover, they appear in the first 8 lines of Table2, which summarizes means and standard deviation of the indices ordered according to the discrimination ranking.

**Table 2:** Means and standard deviations (SD) of individual-specific and universal indices prior to standardization

Discrimination ranking	Index	Mean and SD prior to standardization
*Individual-specific index*		
1	MP3 (Index 24)	2.347 ± 1.341 sec
2	Mean-AP (Index 2)	51.47 ± 18.04 mm
3	Beta-AP (Index 22)	0.11 ± 0.046
4	Zero-cross-V-AP (Index 16)	146.4 ± 20.67
5	Mean-ML (Index 1)	0.742 ± 6.409 mm
6	log-MV-ML (Index 71)	0.686 ± 0.12 mm/sec
7	log-MV-AP (Index 72)	0.798 ± 0.097 mm/sec
8	log-Alpha-ML (Index 19)	0.084 ± 0.074
9	log-Alpha-AP (Index 21)	0.163 ± 0.115
11	Beta-ML (Index 20)	0.14 ± 0.042
12	log-Slope-MP (Index 27)	0.143 ± 0.272 sec/mm
17	log-Power-ML (Index 37)	2.016 ± 0.273 mm^2^/Hz
19	PF95-AP (Index 42)	1.141 ± 0.285 Hz
39	log-LNG (Index 17)	2.792 ± 0.098 mm
–	log-Power (Index 34)	2.001 ± 0.254 mm^2^/Hz
–	log-MV (Index 70)	0.947 ± 0.098 mm/sec
*Universal index*		
–	Angle (Index 6)	62.45 ± 23.5 degree
–	Slope-L-ML (Index 9)	−0.996 ± 0.61 mm^2^/Hz^2^
–	Slope-L-AP (Index 12)	−1.2 ± 0.62 mm^2^/Hz^2^
–	PF50-ML (Index 38)	0.324 ± 0.066 Hz
–	PF50-AP (Index 41)	0.329 ± 0.071 Hz
–	Flattening (Index 73)	0.377 ± 0.165

Index 24 (MP3) has the highest ranking. It represents the mean peak value of the Sway Density Curve (SDC) that characterizes how densely a sway trajectory stays locally in the AP-ML plane as a function of time. More specifically, an SDC represents changes in the time duration of how long CoP trajectory stays locally and time-continuously inside a circle with a radius of *R* mm, centered at a CoP point at every sampling instant of time (Jacono et al. 2004), where *R* = 3 mm for obtaining MP3. It often exhibited an oscillatory waveform for *R* = 3 mm, which means that CoP stays locally for a period of time (corresponding to a peak of the oscillatory SDC waveform), and then migrates to another location (corresponding to a valley of the SDC). See Fig. S3. The MP3 index quantified the mean of peak values of such oscillatory SDC waveforms for *R* = 3 mm. Thus, the larger the values of MP3, longer the time duration the CoP trajectory stays locally. As shown in Table2, the mean of MP3, for the original values prior to the standardization across subjects, was about 2.3 sec with a large subject-dependent variability, during which CoP was trapped locally in a small circle. This means that CoP exhibited an oscillation of small amplitude (less than 3 mm) in a localized area with frequencies higher than 0.43 Hz, roughly corresponding to the fast or the very fast oscillatory components.

Index 2 (Mean-AP) has the second-best ranking. It corresponds to the mean position of CoP-AP.

Index 22 (Beta-AP) has the third best ranking. It represents the scale parameter of Gamma distribution that approximates the distribution of time intervals of changes in the sign of CoP-AP velocity. Such function is formulated as follows:

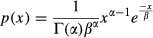
4

where *x* represents the inter-zero-cross interval in the CoP-AP velocity profile, and the parameters *β* and *α* provide the index values of Beta-AP and log-Alpha-AP, respectively. Note that the mean and variance of this distribution are *αβ* and *αβ*^2^, respectively. Histograms for sequences of the inter-zero-cross intervals in the CoP-AP velocity for each CoP time series were fitted by this distribution. See Fig. S4. The distribution of inter-zero-cross intervals in the CoP velocity was introduced in this study based on our consideration such that the fast and the very fast oscillatory components of sway would generate the corresponding inter-zero-cross intervals in the CoP velocity profile. With a Gamma-fitted distribution, large and peaky unimodally distributed small intervals would appear when the CoP velocity profile clearly and predominantly contains the very fast oscillatory components, which can be characterized by small values of Beta-AP combined with large values of log-Alpha-AP. That is, the more frequently and clearly the very fast oscillatory component is contained in the CoP velocity profile, the smaller the values of Beta-AP (*β*) and the larger the values of log-Alpha-AP (*α*) are estimated. Indeed, in the data set analyzed in this study, the mean value of Beta-AP across subjects was small at about 0.1 (Table2), and the median of the Gamma-fitted-distribution was roughly about 0.2 seconds. The inter-zero-cross intervals of 0.2 seconds in the CoP velocity profile corresponds to the oscillation with frequency of about 2.5 Hz, which corresponds to the very fast oscillatory component.

Index 16 (Zero-cross-V-AP) has the fourth best ranking. It represents the number of changes of sign of low-pass-filtered CoP-AP velocity. It was introduced in this study to characterize the low-frequency changes in the sign of CoP-AP velocity, based on our consideration such that the CoP velocity profile might include both of the fast (0.5–1.0 Hz) and the very fast (1.0–2.5 Hz) oscillatory components, and the low-pass-filtering would enhance the former by eliminating the latter. See Fig. S1. Indeed, the mean of the Zero-cross-V-AP index, for the original values prior to the standardization across subjects, was about 140 times in 70 s (Table2), corresponding to the inter-zero-cross-intervals of the low-pass-filtered CoP velocity of about 0.5 seconds and the oscillation in the CoP velocity profile with 1.0 Hz, and thus roughly to the fast oscillatory component.

In order to show there was no over-fitting in the selection of the 8 indices defined above, we analyzed the subject discrimination test by the optimal linear classifier in relation to the set of 8 indices. We found that the apparent error rate was rather small: 9.8%. Also the error rate of leave-one-out cross-validation was small: 11.0%. On the other hand, when the whole set of 40 indices selected by the AIC-based stepwise method was used, the apparent error rate and the error rate of leave-one-out cross-validation were 3.3% and 6.3%, respectively, and this was comparable in performance to the optimal linear classifier.

### VM-VV plot and candidates of universal indices

**Figure 5:**
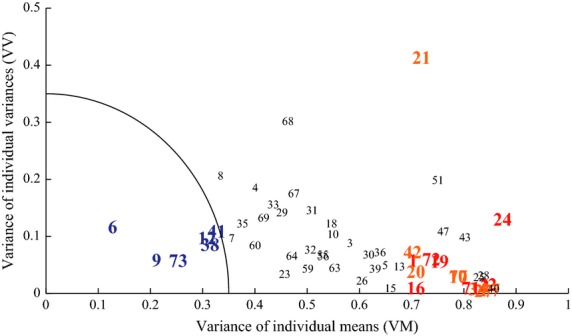
The variance of individual means (VM) and the variance of individual variances (VV) of each index across subjects. Numbers plotted in the VM-VV plane represent the index numbers. The candidates of universal and individual-specific indices were colored in blue and red, respectively. Indices colored in orange were also considered as individual-specific later by the correlation analysis. Indices colored in black were neither universal nor individual specific.

The identification of universal index candidates was performed using the previously defined VM-VV plot, with the criterion that indices closer to the origin can be considered more likely candidates. We chose a distance of 0.35 from the origin as the selection threshold. In Fig.5, such threshold is represented by the circular sector. The whole set of 73 indices is plotted in the figure and it appears that 6 indices fall inside the circular region: 6, 9, 12, 38, 41 and 73. These indices are taken as candidates of the universal group. Most of these candidates are associated with the temporal structure of CoP patterns at the low-frequency regime: index 9 (Slope-L-ML), index 12 (Slope-L-AP), index 38 (PF50-ML), and index 41 (PF50-AP).

Slope-L-ML and Slope-L-AP represent, respectively, the scaling exponents of CoP-ML and CoP-AP at the low-frequency band (between about 0.04 Hz and 0.5 Hz). As shown in lower part of Table2, the mean ± SD values of Slope-L-ML and Slope-L-AP across subjects, for the original values prior to the standardization, were −0.996 ± 0.61 and −1.2 ± 0.62, respectively, and they were roughly consistent with the characteristics associated with the intermittency observed in postural sway (Collins and De Luca 1993) and in an intermittent postural control model (Asai et al. 2009).

PF50-ML and PF50-AP represent the frequencies that bisect the total powers of CoP-ML and CoP-AP, respectively. Thus, the larger the power in the low-frequency side, the lower (the smaller) the PF50 values. The mean ± SD values of PF50-ML and PF50-AP across subjects (the original values prior to the standardization) were 0.324 ± 0.066 Hz and 0.329 ± 0.071 Hz, respectively, meaning that the postural sway of all subjects exhibited low-frequency variability prominently with similar amounts of rate against the total power. In general, it appears that universal features of CoP patterns are associated with the low-frequency slow components of sway.

The remaining candidates are index 6 (Angle of the 95% confidence ellipse of CoP) and index 73 (Flattening of the same ellipse). They are associated with the overall geometry (not detailed structure) of the CoP trajectory in the AP-ML plane. Note that index 6 did not pass the normality test.

As a cross-check, we can see from Fig.5 that indices that were identified by the linear discriminant analysis as individual-specific candidates are located far away from the origin.

**Figure 6:**
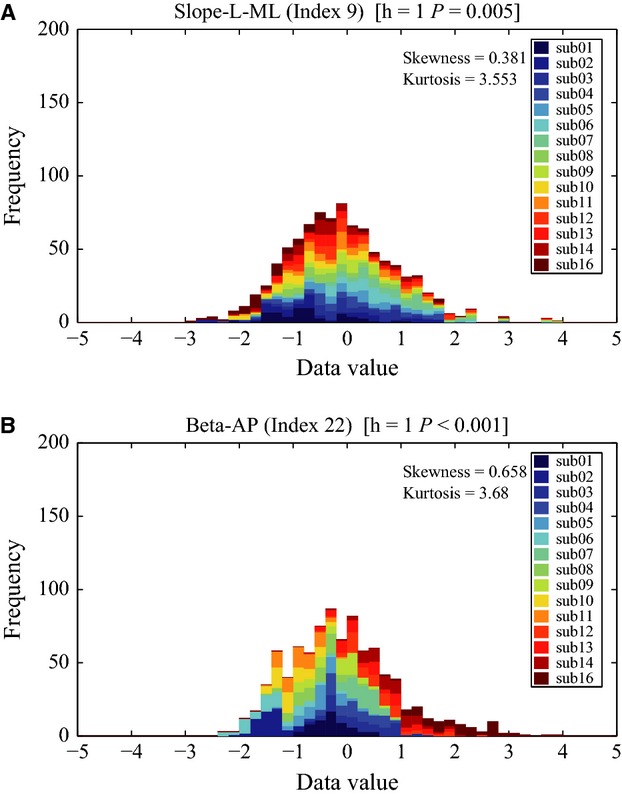
Pooled histograms stacked over all subjects for Slope-L-ML (Index 9) in (A) and for Beta-AP (Index 22) in (B). The title of each panel indicates the *h* and *P*-values of the Lilliefors' test for each index. The index Slope-L-ML was considered as universal, for which the pooled histogram was similar to the normal distribution and each bin of the histogram were almost evenly occupied (stacked) by subject-wise different colors. On the other hand, the index Beta-AP was considered as individual specific, for which shape of the pooled histogram was asymmetry with a long tail and was not similar to the normal distribution. Moreover, each bin of the histogram was not evenly occupied by subject-wise colors.

**Figure 7:**
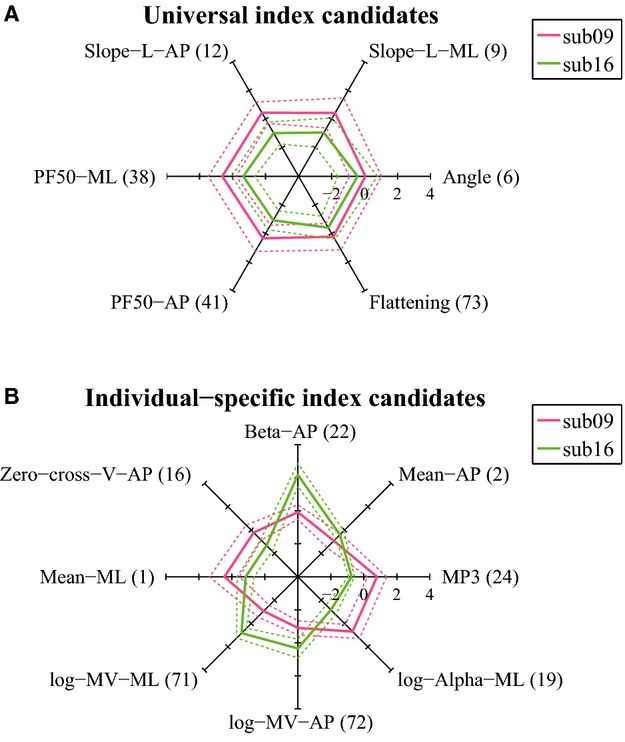
Radar charts illustrating how the CoP time-series of two representative subjects were characterized commonly and differently, respectively, by the set of values of the universal index candidates and by the set of individual-specific index candidates. In each panel, solid lines connect the individual mean values of the indices, and dashed lines connect the individual mean ± SD values of the indices. (A) Universal index candidates. (B) Individual-specific index candidates.

In order to illustrate the properties of the two main classes of indices, namely universal vs. individual indices, let us consider two representative pictures: Figs.6 and 7. Figure6 compares a typical universal index (number 9, Slope-L-ML) and a typical individual index (number 22, Beta-AP). In both cases, the figure shows the pooled histogram of the corresponding index. In the former case, the overall histogram is Gaussian-like and the color-coded histograms of the individual subjects are well aligned with it, a feature that we can expect of a universal index. In contrast, the overall histogram of index 22 is far from Gaussian-shaped and the individual histograms are distributed differently, a typical feature of an individual index.

Figure7 compares the distribution of the two groups of indices for two representative subjects (subject codes 9 and 16). The top panel shows the radar chart of the 6 universal indices (numbered 6, 9, 12, 38, 41, 73), and the bottom panel that of the 8 individual indices (numbered 1, 2, 16, 19, 22, 24, 71, 72). It is evident that in the former case the charts for the two subjects are quite similar, with overlapping range of variation. In the latter case, the charts are quite different, enhancing subject-specific features.

### Improvement, validation and characterization of the classification

**Figure 8:**
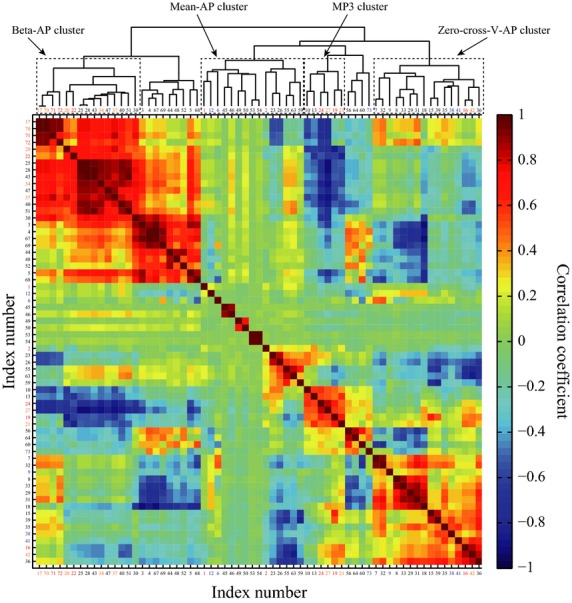
Correlations between two indices for all possible combinations of indices. The panel color at (k,k′)-grid represents the correlation coefficient between k-th and k′-th indices. The indices were rearranged by the dendrogram representing the similarity of pair of two indices. This dendrogram was drawn up on the basis of hierarchical cluster analysis where the correlation coefficients were used as the distance among indices.

Figure8 shows the correlation coefficients for the pairwise correlation analysis among normally distributed 65 indices and the dendrogram obtained by the hierarchical cluster analysis. By the pairwise correlation analysis, we identified eight additional indices that are strongly correlated (absolute values of correlation coefficient greater than 0.8) with the previously defined individual-specific index candidates (indices 24, 2, 22, 16, 1, 71, 72 and 19). The newly added eight indices are the indices 17, 20, 21, 27, 34, 37, 42 and 70. This extends the individual-specific group to 16 elements, as reported in Table2.

It was confirmed that, for the newly added eight individual-specific indices, variances of individual means (VM) across subjects were large, and those of individual variances (VV) across subjects were small. Thus, they were located near the previously determined eight individual-specific index candidates in the VM-VV plot (see Fig.5). This means that those newly added eight indices include information useful for the individual discrimination, but they were redundant due to high correlations with the individual-specific index candidates. Thus, they did not contribute to lowering the AIC in the linear discriminant analysis.

The way of how the extended individual-specific indices correlate with each other could be summarized by the hierarchical cluster analysis (the dendrogram in Fig.8), in which the sixteen individual-specific indices were clustered into a set of four clusters indicated by the dotted rectangles in the dendrogram. Integration of the linear discriminant analysis, the correlation analysis and the hierarchical cluster analysis revealed four major factors (actually three as described below) that were responsible for the characterization of the sixteen individual-specific indices. The linear discriminant analysis clarified the indices 24, 2, 22, 16 and 1, in this descending order for the ability of individual discrimination. The fact that the first four indices (24, 2, 22, and 16) were clustered into the four distinguished groups implied that they characterized different aspects of sway.

The first major factor was represented by the most individual-specific index 24 (MP3), to which the individual-specific indices 19 (log-Alpha-ML), 21 (log-Alpha-AP) and 27 (log-Slope-MP (Jacono et al. 2004)) were clustered together, referred to as the MP3 cluster, by the positive correlations with MP3. As defined by equation 4, the indices 19 (log-Alpha-ML) and 21 (log-Alpha-AP) represent the shape parameters of Gamma distributions, which were dominantly determined by the very fast oscillation in the velocity profile with frequency of 2.5 Hz, together with the indices 20 (Beta-ML) and 22 (Beta-AP) that were grouped in the third cluster as described below. Index 27 (log-Slope-MP) is directly related to index 24 (MP3), since it represents how the MP values change as the radius *R* of local circle increases. Since index 24 (MP3) represents the sway power with frequencies higher than about 0.43 Hz as described above, all of these indices were associated with the fast and the very fast oscillatory components of sway. The positive correlation between MP3 and log-Alpha indices could be described as follows: (1) The larger the values of MP3, the longer the time durations of sustained small amplitude oscillation within the local circles. (2) The more frequently and clearly the very fast oscillatory component is contained in the CoP velocity profile, the smaller the values of Beta-AP (*β*) and the larger the values of log-Alpha-AP (*α*) are estimated as described above. Since the appearance of sustained small amplitude oscillation might correspond to the very fast oscillatory component, the larger the values of MP3, the larger the values of log-Alpha-AP, yielding the positive correlation between MP3 and log-Alpha-AP. See Fig. S4. Note that this logic was reflected in the negative correlation between MP3 and Beta-AP.

The second factor was represented by the second most individual-specific index 2 (Mean-AP), to which the fifth best discriminant individual-specific index 1 (Mean-ML) was clustered, referred to as the Mean-AP cluster. These two indices could be considered as a measure of motor habit, which vary depending on if a subject tended to stand with droopy posture or with backward inclining posture, and with left or right inclining posture.

The third factor was represented by the third best discriminant individual-specific index 22 (Beta-AP), to which the five newly added individual-specific indices (indices 20, 34, 37, 17 and 70) and two previously selected individual-specific indices 71 and 72 were clustered together, referred to as the Beta-AP cluster, by the positive correlations with Beta-AP. One could observe that the Beta-AP cluster was composed of two sub-clusters {20, 22, 34, 37} and {17, 70, 71, 72}. Index 20 (Beta-ML) and index 22 (Beta-AP) was positively correlated because Beta-ML also characterized the very fast oscillation with frequency about 2.5 Hz as in Beta-AP. Indices 34 and 37 in the first sub-cluster represent log-Power and log-Power-ML, respectively. Those CoP-power-related indices and Beta-AP (and Beta-ML) were positively correlated by the following complicated logic. Firstly, an increase in Beta-AP implies lowering of a value of the single modal peak in Gamma distribution of CoP velocity profile and widening of the peak, where a large value of the single modal peak corresponds to frequent appearances of small inter-zero-cross intervals. Thus the lowering of the peak value means diminishing of the very fast component (see eq. 4). Although the diminishing of the very fast component resulted in lowering in CoP-power at high- frequency regime, actually it caused an overall increase in CoP-power at high- frequency regime. This was because CoP amplitude accompanied by the very fast velocity component was small (less than 3 mm), and the diminishing of the very fast component could not much affect the CoP-power. Instead, decreases in the occurrence of small inter-zero-cross intervals (<0.2 sec; the very fast component) resulted in increases in the occurrence of larger inter-zero-cross intervals than 0.2 sec (but smaller than 1.0 sec), which contributed to the increase of CoP-power in the frequency range of about 0.5–1.0 Hz, because CoP amplitude accompanied by the changes in CoP velocity in this frequency range could be much larger than that in the frequency of the very fast component. In this way, Beta-AP cluster characterized essentially the same aspect of CoP pattern as the MP3 cluster, i.e., the very fast component of sway, although these two clusters were distantly located in the dendrogram, due to the negativity of the high correlation between Beta-AP and MP3.

Indices 17, 70, 71 and 72 in the second sub-cluster of the Beta-AP cluster represent log-LNG (the total path length of CoP trajectory on AP-ML plane), log-MV (the mean velocity of CoP of planar movement), log-MV-ML and log-MV-AP, respectively, and all of them were associated directly with the mean sway velocity. Under the situation that the low-frequency power (PF50) was almost invariant and independent of the subjects, the increase (decrease) in the mean sway velocity could be caused only by the increase (decrease) in the fast and the very fast oscillatory components, resulting in the positive correlations between the indices in the second sub-cluster and Beta-AP.

The fourth factor was represented by the fourth best discriminant individual-specific index 16 (Zero-cross-V-AP), to which the newly added index 42 (PF95-AP) was clustered, referred to as the Zero-cross-V-AP cluster, by the positive correlation between them. Note that PF50-ML and PF50-AP were selected as the universal indices, whereas PF95-AP was selected as the individual-specific index. Since the mean value of PF50-AP across subjects was about 0.32 Hz, and the mean value of PF95-AP was about 1.1 Hz (see Table2), the subject specificity (subject-dependent feature of the PSD) characterized by PF95-AP was due to difference in the shape and power of PSD at the frequency band between 0.32 Hz and 1.1 Hz, at the middle of which typical frequency of the fast oscillatory component (0.6 Hz) is located.

Correlation coefficients between each of the six universal index candidates and all the remaining indices were also calculated. In no case the correlation was greater than the chosen threshold of 0.8, thus the group of universal indices was not extended by the correlation analysis.

Finally, we used the linear mixed-effect model for validating the classification in the two groups. Table3 reports the quantification of factors of the variances for both groups of indices. It appears that for all the universal index candidates (indices 6, 9, 12, 38, 41 and 73) the ratios *σ*_sub_/*σ*_e_ are less than 1.0, confirming that, as expected, the major factor that causes variability of the universal index candidates is not subject, but the neutral errors. In contrast, all the individual-specific indices have values of the ratio *σ*_sub_/*σ*_e_ which are largely greater than 1.0, confirming that, as expected, the major factor that causes variability of the individual-specific indices is subject.

**Table 3:** Ratio of subject-depend variation to residual of mixed effect model for the universal and the individual-specific indices

Discrimination ranking	Index	Ratio of subject variation to residual
*Individual-specific index*		
1	MP3 (Index 24)	5.054
2	Mean-AP (Index 2)	4.615
3	Beta-AP (Index 22)	5.407
4	Zero-cross-V-AP (Index 16)	2.419
5	Mean-ML (Index 1)	2.395
6	log-MV-ML (Index 71)	4.295
7	log-MV-AP (Index 72)	3.066
8	log-Alpha-ML (Index 19)	3.004
9	log-Alpha-AP (Index 21)	2.701
11	Beta-ML (Index 20)	2.43
12	log-Slope-MP (Index 27)	4.652
17	log-Power-ML (Index 37)	4.067
19	PF95-AP (Index 42)	2.158
39	log-LNG (Index 17)	4.102
–	log-Power (Index 34)	2.893
–	log-MV (Index 70)	4.102
*Universal index*		
–	Angle (Index 6)	0.113
–	Slope-L-ML (Index 9)	0.246
–	Slope-L-AP (Index 12)	0.409
–	PF50-ML (Index 38)	0.881
–	PF50-AP (Index 41)	0.625
–	Flattening (Index 73)	0.302

### Correlation between body parameters and the two groups of indices

**Figure 9:**
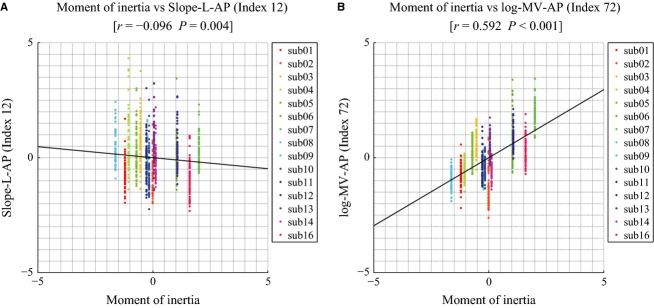
(A) Scatter plot of Slope-L-AP (Index 12) as one of the universal index versus the moment of inertia of each subject. (B) Scatter plot of log-MV-AP (Index 72) as one of the individual-specific index versus the moment of inertia of each subject. Individual-specific index tended to correlate more with the moment of inertia than universal index.

We may expect that an index of the universal group is uncorrelated or very weakly correlated with a body parameter as the moment of inertia of a subject. At the same time we may expect the opposite for an index of the individual-specific group. Figure9, which shows the scatter plots of index 12 (universal group) in panel A and the plot of index 72 (individual-specific group) in panel B, confirms this relationship.

Moreover, Table4 reports the correlation coefficients with the moment of inertia for all the indices of the two groups. It appears that all the indices of the universal group have a very small correlation, in any case smaller than 0.1. For the indices of the individual-specific group we have a different pattern: almost half of them are somehow correlated (indices 17, 27, 34, 37, 70, 71, and 72; correlation value greater than 0.4); three of them are uncorrelated (indices 2, 19, 21; correlation value less than 0.1); the remaining indices (1, 16, 20, 22, and 42) are weakly correlated.

**Table 4:** The correlation coefficients between the moment of inertia and the index values, separately for the universal and the individual-specific indices

Discrimination ranking	Index	Correlation coefficient
*Individual-specific index*		
1	MP3 (Index 24)	−0.332
2	Mean-AP (Index 2)	−0.06
3	Beta-AP (Index 22)	0.303
4	Zero-cross-V-AP (Index 16)	0.149
5	Mean-ML (Index 1)	−0.216
6	log-MV-ML (Index 71)	0.571
7	log-MV-AP (Index 72)	0.592
8	log-Alpha-ML (Index 19)	−0.028
9	log-Alpha-AP (Index 21)	0.08
11	Beta-ML (Index 20)	0.324
12	log-Slope-MP (Index 27)	−0.478
17	log-Power-ML (Index 37)	0.404
19	PF95-AP (Index 42)	0.186
39	log-LNG (Index 17)	0.629
–	log-Power (Index 34)	0.413
–	log-MV (Index 70)	0.629
*Universal index*		
–	Angle (Index 6)	−0.056
–	Slope-L-ML (Index 9)	0.039
–	Slope-L-AP (Index 12)	−0.096
–	PF50-ML (Index 38)	0.086
–	PF50-AP (Index 41)	0.035
–	Flattening (Index 73)	−0.041

Finally, Wilcoxon rank sum test was carried out to examine whether the absolute values of correlation coefficients between the universal indices and the moment of inertia and those between the individual-specific indices and the moment of inertia were significantly different. The results show that there are significant differences with *P* < 0.01, indicating that values of most of the individual-specific indices depended on the body parameter of each subject, whereas values of the universal indices were not determined by the body parameters.

## Discussion

The main aim of this study was to identify, in the sway patterns of healthy young subjects during quiet standing, universal features, common to all subjects and independent of individual anthropometric parameters as well as circadian biological rhythms. The rationale is that such universal features may reflect a common motor control strategy adopted by the central nervous system of any individual for stabilizing the upright posture. A complementary aim of the study was to distinguish such universal features, captured by a number of indices, from individual-specific features.

In order to achieve such goals, a large number of CoP time-series were collected from a population of healthy young subjects. From this dataset a redundant set of indices (seventy three of them, initially) was extracted. An articulated statistical analysis of the indices allowed us to sort out a subset of six universal indices and a subset of eight plus extended eight individual-specific indices.

Our analysis showed that the universal indices characterize mainly the slow component of sway, such as scaling exponents of power-law behavior at a low- frequency regime (0.04–0.5 Hz). On the other hand, it was shown that the individual-specific indices were associated with the fast (0.5–1.0 Hz) and the very fast (1.0–2.5 Hz) oscillatory components and the mean CoP position on the ML-AP plane. Moreover, we showed that the individual-specific indices were more significantly correlated with body parameters than the universal indices, implying that biomechanics of each individual, rather than neural control, characterizes the individual-dependent features of postural sway. In this section, we discuss how our statistical index classification can be interpreted, that is, how the indices associated with the fast/very fast and the slow dynamics of sway were classified as individual-specific and universal, respectively.

### Robustness of the statistical classification

Before addressing physiological interpretations of our results, here we discuss robustness of our statistical classification, i.e., how our assumptions, numbers for decision criteria used in our statistical analysis would affect the results.

#### Criterion for normality test

In this study, we used a criterion to pass the normality test for each index such that intrasubject set of values of the index is normally distributes for more than 70% of all subjects. We have confirmed that other threshold values for this criterion, including 80% and 60% for example, did not alter the main result of the classification. More specifically, the use of 80% resulted in three additional indices (Indices 41, 43 and 51) included in the set of normally distributed indices, and the use of 60% excluded three indices (Indices 38, 45 and 47) from the set of normally distributed indices. However, those six affected indices have not been included in the candidates of individual-specific index, and thus we conclude that the results of our classification are independent of this threshold parameter.

#### The threshold for selecting the candidates of universal index

We used the threshold value (the threshold distance from the origin of the plane on the VM-VV plane) 0.35 to select six candidates of the universal index. We have confirmed that other threshold values, including 0.3 and 0.4 for example, did not alter the main result of the classification. More specifically, the use of 0.3 as the threshold value excluded three indices (indices 12, 38 and 41) from the universal indices. However, the index 9, one of the remaining three universal indices (indices 6, 9 and 73), still characterizes the slow sway component, which causes no changes in our physiological interpretation of the classification. The use of 0.4 as the threshold value included three additional universal indices (indices 7, 8 and 35) into the universal group. However, the indices 7 (Mean-cross-ML) and 8 (Mean-cross-AP) characterize the numbers of CoP zero-crossing, and their values were about 40, corresponding to the frequency of CoP motion with about 0.3 Hz that falls in the range of the slow component. Moreover, Index 35 represents PF50 that also characterizes the slow component. Thus, once again, the use of threshold value 0.4 did not cause any change in our physiological interpretation. In this way, the main result of this study shows robustness against the choice of the threshold value for selecting the universal indices in the VM-VV plane.

#### The threshold for the correlation analysis

We used the threshold value 0.8 for selecting indices that were correlated with the candidates of individual-specific indices to improve our classification. We have confirmed that other threshold values, including 0.9 and 0.7 for example, did not alter the main result of our classification. More specifically, the use of 0.9 as the threshold value excluded six indices (indices 20, 21, 27, 34, 37 and 42) from the set of revised individual-specific indices. All of them characterize the fast and/or the very fast components of sway. However, the remaining set of individual-specific indices still include those representing the fast and the very fast components of sway, and thus the use of threshold value 0.9 did not cause any change in our physiological interpretation. Similarly, the use of 0.7 as the threshold value included eight additional indices (Indices 25, 28, 30, 36, 43, 47, 55 and 63) into the individual-specific group. However, once again, all of them characterize the fast and/or the very fast components, which causes no changes in the main result of this study.

### Slow and fast sway dynamics

We start our discussion by focusing on specific frequency components of sway patterns during quiet standing. It has been clarified that each of CoP-ML and CoP-AP patterns contain three characteristic frequency components: a slow (either nonoscillatory or oscillatory) component (Kiemel et al. 2006; Nomura et al. 2013), a fast damped-oscillatory component (Kiemel et al. 2006), and a very fast oscillatory component representing the anti-phase coordinated trunk-leg movement (Creath et al. 2005; Suzuki et al. 2012). Zatsiorsky and Duarte (Zatsiorsky and Duarte 1999) termed the slow and the fast components (∽0.16 Hz and ∽0.67 Hz, respectively, for their experimental subjects) as “rambling” and “trembling”, respectively. Creath et al. (2005) showed that the trunk and the leg segments during quiet standing exhibited antiphase oscillation particularly in the frequency above 1 Hz (1.0–5.0 Hz), which was predominantly related to the mechanical characteristics of a double-inverted-pendulum-model of the standing body. We deemed such antiphase oscillations as the very fast components highlighted in this study. The main contribution of this study is that our purely statistical analysis revealed one-to-one correspondences between those mechanistically interpreted three types of sway dynamics (one associated with the slow component, the other two associated with the fast and the very fast components) and our statistically classified three types of sway indices. More specifically, the slow, the fast and the very fast components might correspond, respectively, to the major factor characterizing the universal indices and the two major factors characterizing the individual-specific indices.

The mechanism of how the fast damped-oscillatory component is generated can be understood simply by considering the standing body as an inverted pendulum. In this simple model, the upright posture is unstable equilibrium point of saddle-type, with stable and unstable modes, i.e., stable and unstable manifolds (Bottaro et al. 2008; Asai et al. 2009). Feedback control is thus indispensable for stabilizing the upright equilibrium, for which a proportional-derivative feedback controller (with feedback delay time) has been considered in most previous studies (Johansson et al. 1988). If a gain of the derivative feedback controller is not too large relative to a gain of the proportional feedback controller, then the system exhibits damped-oscillatory dynamics with small amplitude. Typical frequency (resonant frequency) of this oscillation is faster than the remaining major slow component within a range of frequency 0.5–1.0 Hz (Kiemel et al. 2006) or 0.67 Hz in the study of Duarte et al. (Zatsiorsky and Duarte 1999). Since the frequency of the fast oscillatory component is determined by the individual body parameters, it is reasonable for the indices that characterize the fast oscillation in the range of 0.5–1.0 Hz to be classified as individual-specific in this study.

Recent studies have revealed double-inverted-pendulum-like behaviors during quiet standing (e.g., Creath et al. 2005; Hsu et al. 2007; Sasagawa et al. 2014). It is becoming a common view that the ankle and hip strategy (Horak and Nashner 1986) are implemented not only in response to perturbations, but also during quiet stance along with a mixed strategy (Creath et al. 2005; Suzuki et al. 2012). Spectral analysis of powers and co-phases of trunk and leg movement (Creath et al. 2005) and eigenfrequency analysis of mechanical, nonactively-controlled double-inverted-pendulum model (Suzuki et al. 2012) during quiet standing have shown that in-phase and antiphase oscillations between upper and lower links with about 0.5 Hz and 1.5–2.0 Hz, respectively. Those evidences support the idea that neural control may not be the primary determinant of the coordinated postural sway patterns, but they might arise from the biomechanical dynamics of a double-(multi-link)-inverted-pendulum-like body. Since these frequencies of in-phase and antiphase coordinated patterns are determined by the individual body parameters (including parameters for multiple links as well as passive joint viscoelasticity), the indices that characterize the fast oscillation in the range of 0.5–1.0 Hz, which might correspond to the in-phase coordination, and the very fast oscillation in the range of 1.0–2.5 Hz, which might correspond to the antiphase coordination, were classified as individual-specific in this study.

The slow component can be considered as either nonoscillatory (Kiemel et al. 2006) or oscillatory (Nomura et al. 2013). However, in either case, it might be associated with exponential decay back to equilibrium while being continually perturbed by noise during quiet upright stance. It accounts for the majority of sway variance, and this fact is characterized by the indices PF50-ML and PF50-AP (the universal indices 44 and 47). That is, the larger the power of slow dynamics, the smaller the values of PF50 as mention in the result section. Thus, these two universal indices are associated with the power of the slow dynamics, though indirectly.

Mechanisms of how the slow component is generated are less understood. Zatsiorsky and Duarte (1999) hypothesized that the slow dynamics are due to a slowly-migrating reference point defined by a central command. Kiemel et al. (2002, 2006) hypothesized that the slow dynamics are caused by estimation errors of the postural state. The origin of slow dynamics in one type of intermittent control proposed by Loram et al. (2011) might be similar to the one by Kiemel et al., but Loram et al. consider that the state estimations and resulting neural interventions are carried out intermittently by the central nervous system. An alternative view of the slow dynamics has been provided by the double-power-law behavior of postural sway at low and high-frequency regimes, which are considered to be associated, respectively, with closed-loop and open-loop postural control (Collins and De Luca 1994). The power-law behavior at the low-frequency regime is characterized by the universal indices as the indices Slope-L-ML and Slope-L-AP (indices 9 and 12), meaning that these two universal indices quantify the slow dynamics. There are several versions of mathematical models, referred to as intermittent control models, which can account for the double-power-law behaviors at the low-frequency regime (Collins and De Luca 1993; Bottaro et al. 2008; Asai et al. 2009; Milton et al. 2009; Suzuki et al. 2012; Nomura et al. 2013). Although the models by Collins and De Luca or by Milton et al. and the other models assume substantially different mechanisms of stabilization, both of them consider basically that the switching between open-loop and closed-loop dynamics are responsible for the double-power-law behaviors at the low-frequency regime.

In this way, the indices quantifying slow dynamics, including Slope-L-ML and Slope-L-AP (indices 9 and 12), were identified as universal, suggesting that the neural control inherent for stabilizing upright posture is expressed as the universal characteristics of postural sway as we expected at the beginning of this study. As Kiemel et al. (Kiemel et al. 2006) stated, characterizing the slow process may hold the key to determining the underlying basis of balance problems in populations with poor balance control. Note, however, the current study cannot provide any evidence that is useful for arguing which model is more physiologically plausible as a neural control strategy employed by the central nervous system. What is interesting of this study is that nonmechanistic, purely statistical analysis of a set of sway data could reach the sway-index-classification that is quite consistent with the mechanistic interpretations on the origins of postural sway.

### Remarks and limitations of the current study

The first remark is associated with the characterization of sway using the sway area. In this study, the index log-Area (index 3) characterizing sway area and the index RMS (indices 67, 68 and 69) were classified into neither universal nor individual-specific indices. This means that the sway area and RMS are not much informative, neither for characterizing neural control nor for subject-dependent biomechanics during quiet standing in healthy young subjects. This is rather surprising because these indices are very popular and widely used in research and clinical applications for evaluating postural stability (e.g., Krishnamoorthy et al. 2002; Raymakers et al. 2005; Seigle et al. 2009).

There are two possible explanations of this counter-intuitive result. One explanation may be attributed to the limitation of the population of subjects addressed by this study, namely healthy young men, and specific features of the experimental protocol such as the fact that only the open-eyes condition was considered. That is, the homogeneity of subjects and the single-standing condition might have eliminated age-dependency (Seigle et al. 2009), standing-condition-dependency (Raymakers et al. 2005), and disease-induced changes (Schieppati et al. 1994) of sway area. Thus, the identification of universal and individual-specificity indices performed by this study should be strictly applied only to the healthy young adults, although an extension of the method to other populations could usefully take our results as an informative starting point.

The other reason is associated with the reliability (robustness) of sway area (and also RMS) estimation. Indeed, the sway area might be individual-specific per se, rather than universal as can be observed by the fact that the location of Index 3 point in the VM-VV plane (Fig.5) is relatively closer to the area for the individual-specific indices than that for the universal indices. However, for the purpose of individual characterization, sway area measured by 95% confidence ellipse might not be suitable. This is because determination of 95% confidence ellipse is too sensitive to large excursions that take place rarely in CoP trajectory. For example, suppose a case in which a subject exhibiting small sway area for most of the standing trials shows a single event of large excursion deviated away from the usual sway area incidentally. Such a rare event may occur only once in a single trial or even rarely in multiple trials. Despite the rareness of such events, it can easily make the area of 95% confidence ellipse large, which makes the individual variances (intrasubject variances) bigger, hindering the index of sway area from being the individual-specific. In fact, the indices of log-Power and log-Power-ML (Indices 34 and 37) quantifying the sway power between 0.15 and 5.0 Hz were identified as the individual-specific, despite of the fact that these are the frequency-domain correspondences of the sway area, where the band-limited integration might filter out the power of rare events.

The second remark is associated with the indices of Angle and Flattening that were identified as universal, other than the slow-component-related indices. These two indices characterize the shape of 95% confidence ellipse, and universality of these two indices might reflect the fact that the CoP tends to fluctuate in the AP direction, rather than in the ML direction, in most of the subjects. However, 95% confidence ellipse might not be robust for the same reasons proposed above. Thus these two universal indices might not be as informative as the slow-component-related universal indices.

The third remark is about the indices associated with the mean CoP velocity, including log-LNG (Index 17), log-MV (Index 70), log-MV-ML (Index 71), and log-MV-AP (Index 72). All of these indices were classified as individual-specific indices. It is worth mentioning that, as shown in Table4, the correlation coefficients between each of these indices and the moment of inertia were quite high, about 0.6. In particular, the correlation was highest for log-LNG and log-MV at about 0.63. This result implies that the indices associated with the mean CoP velocity are merely representing body parameters of subjects (Teasdale et al. 2007). That is, the heavier the weight and the taller the height, the larger the values of these indices, despite of the fact that these indices have often been employed in a number of neurophysiological and neurological studies.

Finally, it is interesting to mention an additional experiment that was carried out, in which some of the subjects were recruited for additional sway measurements during one day (4 trials at 5 different times) about two months after the experiments analyzed in this study. In this additional experiment, 20 CoP data were acquired from each subject. We used indices values calculated from these additional data as novel test data, and examined whether they could be correctly discriminated by the optimal linear classifier obtained in the current study. The error rates for this test were quite low (smaller than about 20%) for all the participating subjects. This means that the individual-specific features of sway are likely to persist at least on a mid-term time horizon.

## Conflict of interest

We declare that there is no conflict of interest.

## (A1)Appendix

### The linear classifier for individual discrimination

Configuration of the linear discriminant functions is formulated as follow. For a set of *q* indices, values of the *k*-th index included in the *q* indices for trial *i* from subject-*p* was denoted here by , (*i* = 1, 2, ···, *n* = 60). The *q*-dimensional index vector for the *i*-th trial in subject-*p*, ***z***_*i,p*_(*q*), is denoted by

5

where *k*(1), *k*(2), ···, *k*(*q*) represent the index numbers of the *q* selected indices. The individual sample mean vector of *q*-dimensional vectors for subject-*p* is denoted by . The total sample mean vector of *q*-dimensional vectors across subjects is denoted by . The individual sample covariance matrix of *q*-dimensional vectors for subject-*p* is denoted by ***S***_*p*_(*q*). The total sample covariance matrix of *q*-dimensional vectors across subjects is denoted by ***S***(*q*) and is calculated as follows:

6

7

8

9

The sum of squared within-group (intrasubject) deviations, denoted by ***W***(*q*), represents the degree of variability of the individual sample ***z***_*i,p*_(*q*) with respect to the corresponding individual sample mean vector. The sum of squared between-groups (intersubject) deviations, denoted by ***B***(*q*), represents the degree of separation between individual sample mean vectors of any two subjects. The total sum of squared deviations from the total sample mean vector across subjects, denoted by ***T***(*q*), represents the variance of *q*-dimensional index vectors (total variation). These matrices are defined as follows

10

11

12

The total variation ***T***(*q*) can then be represented as the sum of ***W***(*q*) and ***B***(*q*):

13

The (*q*−1)-dimensional hyperplane, or linear discriminant function, which optimally discriminates each individual, can be obtained by maximizing ***B***(*q*) and minimizing ***W***(*q*). By equation 13 and the fact that ***T***(*q*) is constant for the given set of data, determining the discriminant function that minimizes ***W***(*q*) is equivalent to determining the discriminant function that maximizes ***B***(*q*). Therefore, the optimal linear discriminant function can be obtained by maximizing the ratio between ***B***(*q*) and ***W***(*q*) or minimizing ***W***(*q*).

For a given *q*-dimensional index vector ***z***(*q*) to be discriminated as either from subject-*p* or subject-*p*′, the optimal linear function that discriminates subject-*p* from subject-*p*′is denoted by , and it can be expressed as

14

In this way, the (*q*−1)-dimensional hyperplane is parameterized by ***W***(*q*) (Rencher 1992). If , the index vector **z**(*q*) was identified as the one from the subject-*p*. If , otherwise, ***z***(*q*) was identified as the one from the subject-*p*′. The optimal linear function that discriminates any combination of *p* and *p*′ was obtained similarly. The linear classifier that can classify any given *q*-dimensional index vector into one of 15 subjects correctly as much as possible was obtained as a set of such optimal discriminant functions.

## (A2)Appendix

### The AIC-based stepwise method

#### Step 1. Obtain a linear classifier using *q* indices

The linear discriminant functions that use a tentatively selected set of *q* indices are calculated, in which the optimal linear classifier  that discriminates subject-*p* from subject-*p*′ for a novel sample index vector **z**(*q*) is given by equation 14. In the case of *q *= 1 for initial setting of the iteration, an index z^(*k*(1))^ is selected arbitrarily from *k*(1) = {1, 2, ···, *M*} where *M* is the number of normally distributed indices, and the linear discriminant functions  are obtained.

#### Step 2. Add a new useful index to the current *q* indices

A new index *z*^(*k*(*q *+ 1))^ that has not been included in the member of *q*-dimensional index vector is selected, and introduced into the current *q* indices to define a new set of (*q *+* *1) indices. For the new (*q *+* *1) indices, the optimal linear classifier is obtained as  for discriminating a novel (*q *+ 1)-dimensional sample index vector ***z***(*q* + 1) = (*z*^(*k*(1))^, *z*^(*k*(2))^, …, *z*^(*k*(*q*))^, *z*^(*k*(*q*+1))^)^*T*^ as in equation 14. The AIC of the linear classifier that optimally discriminates arbitrary combinations of two subjects by the set of *q* indices is denoted by

15

and the amount of change in the values of AIC caused by the addition of z^(k(*q*+1))^ can be expressed as

16

where |A| representing the determinant of a matrix A. The inequality ΔAIC(*z*(*k*^(*q *+ 1))^) > 0, i.e., decrease in the AIC value, means improvement in the performance of the linear classifier by the addition of z^(*k*(*q *+ 1))^, whereas ΔAIC(z^(k(*q *+ 1))^) < 0 means pejoration in the performance. ΔAIC(*z*^(*k*(*q *+ 1))^) are examined for all possible selections of *k*(*q *+* *1). The index *z*^(k(*q *+ 1))^ that takes a positive and maximal value of ΔAIC(*z*^(k(*q *+ 1))^) among the examined *k*(*q *+* *1), if it exists, is employed as the (*q *+ 1)-th index used for the linear discriminant analysis. In this case, Step 2 is completed, and proceed to Step 3. If the maximum value of obtained ΔAIC(*z*^(*k*(*q *+ 1))^) cannot be positive for any possible choice of *z*^(k(*q *+ 1))^, the current set of *q* indices is considered as the optimal, and proceed to Step 4, where the selection of indices completes.

#### Step 3. Eliminate a useless index

An index denoted by *z*^(del)^, except the newly added index *z*^(*k*(*q *+ 1))^ in Step 2, is selected, and eliminated from the current set of (*q *+ 1) indices. The amount of change in AIC value by the elimination of z^(del)^, denoted by ΔAIC(*z*^(del)^), is calculated in a similar way to Step 2. ΔAIC(*z*^(del)^) is evaluated for all possible selections of *z*^(del)^. The index *z*^(del)^ that takes a negative and minimum value of ΔAIC(*z*^(*k*(*q *+ 1))^) among the examined indices, if it exists, is eliminated from the current set of (*q *+ 1) indices, and proceed back to Step 2. If the minimum value of ΔAIC(*z*^(*k*(*q *+ 1))^) cannot be negative for any choice of eliminated indices, no indices are eliminated, and proceed back to Step 2.

#### Step 4. Complete the indices selection

Selection of indices for determining the optimal linear classifier completes.

## Supporting Information

Figure S1 . Definition of Zero-cross-V-ML and Zero-cross-V-AP. **Figure S2**. Zero-crosses of CoP velocity and Gamma-distribution ftting of the inter-zero-cross intervals. **Figure S3**. An example of Sway Density Curve (SDC) with *R *= 3 mm. **Figure S4**. Comparison between SDC and CoP-ML/AP velocity profiles.
