# Supplementary material for: Universal and individual characteristics of postural sway during quiet standing in healthy young adults
Source: Physiol Rep. 2015 Mar 16;3(3):e12329. doi: 10.14814/phy2.12329 (PMC4393163; doi:10.14814/phy2.12329)
Supplement: Figure S1 — . Definition of Zero-cross-V-ML and Zero-cross-V-AP. Figure S2. Zero-crosses of CoP velocity and Gamma-distribution ftting of the inter-zero-cross intervals. Figure S3. An example of Sway Density Curve (SDC) with R = 3 mm. Figure S4. Comparison between SDC and CoP-ML/AP velocity profiles. [file phy20003-e12329-sd1.pdf]

Supplemental Materials

prepared for

Universal and Individual Characteristics of Postural Sway during Quiet Standing in Healthy Young Adults

Tomohisa Yamamoto[a], Charles E. Smith[b], Yasuyuki Suzuki[a], Ken Kiyono[a], Takao Tanahashi[c], Saburo Sakoda[d], Pietro Morasso[e], Taishin Nomura[a,*]

[a] *Graduate School of Engineering Science, Osaka University, Toyonaka, Osaka, Japan*
[b] *Department of Statistics, North Carolina State University, Raleigh, NC, USA*
[c] *Department of Neurology, Osaka University Graduate School of Medicine, Osaka, Japan*
[d] *Department of Neurology, Toneyama National Hospital, Osaka, Japan*
[e] *RBCS Department, Fondazione Istituto Italiano di Tecnologia, Genoa,Italy*

1. Definitions of sway indices

In this section, definitions of the 73 sway indices used in this study are summarized. For each CoP data (low-pass filtered using the fourth-ordered zero-phase-lag Butterworth filter with the cuttoff frequency 10 Hz) during quiet stance of 70 seconds, a value of each of 73 indices was calculated. In this sequel, $\mathrm{CoP}_{\mathrm{org}}$, $\mathrm{CoP\text{-}AP}_{\mathrm{org}}$, and $\mathrm{CoP\text{-}ML}_{\mathrm{org}}$ represent such filtered CoP of planner movements, CoP in Anterior-Posterior (AP) direction and CoP in Medio-Lateral (ML) direction, respectively. The subscript "org" is used to distinguish each of those time-series from the corresponding time-series after taking zero-mean operations. The CoP-ML, CoP-AP and CoP of planner movements after taking the zero-mean operations are simply denoted by CoP, CoP-ML and CoP-AP, respectively, as defined after introducing the indices of Mean-ML and Mean-AP.

*Corresponding author
Address: Graduate School of Engineering Science, Osaka University, Toyonaka, Osaka 5608531 Japan Tel: +81-6-6850-6532, Fax:+81-6-6850-6534, Email:taishin@bpe.es.osaka-u.ac.jp

Mean-ML and Mean-AP (Indices 1 and 2)

The indices Mean-ML and Mean-AP represent the mean positions of CoP-ML and CoP-AP on a force platform coordinate, respectively. By the standing condition that uses a V-shaped guide on the platform (see main text), the origin of the force platform almost coincides with the central point between both internal malleolus. These indices are defined as follows.

$$\begin{aligned}\text{Mean-ML} &= \frac{1}{N}\sum_{n=1}^{N}\text{CoP-ML}_{\text{org}}[n],\\ \text{Mean-AP} &= \frac{1}{N}\sum_{n=1}^{N}\text{CoP-AP}_{\text{org}}[n],\end{aligned}$$

where $[n]$, in this sequel, represents the sampling number. Mean-ML and Mean-AP characterize the degrees of bias of the standing posture from the vertically upright stance in ML and AP directions, respectively. Vuillerme et al. (2002) reported that Mean-AP became large (forward-inclined) when fatigue is accumulated in gastrocnemius [1]. Kirby et al. (1987) reported that Mean-ML and Mean-AP did not change when the degree between both toes became large (duckfooted), but Mean-AP became large when the foot position became toe-in (pigeon-toed) [2].

The CoP-ML, CoP-AP and CoP of planner movements after taking the zero-mean operations are simply denoted by CoP, CoP-ML and CoP-AP, respectively, which are defined as follows;

$$\begin{aligned}\text{CoP-ML}[n] &= \text{CoP-ML}_{\text{org}}[n] - \text{Mean-ML},\\ \text{CoP-AP}[n] &= \text{CoP-AP}_{\text{org}}[n] - \text{Mean-AP},\\ \text{CoP}[n] &= \Big(\text{CoP-ML}[n],\ \text{CoP-AP}[n]\Big).\end{aligned}$$

log-Area (Index 3)

The index log-Area represents the log of the area of 95 % confidence ellipse that encloses 95 % of the points of the CoP path of planar movements. The log-Area is calculated as follows: First, the covariance matrix C of CoP$[n]$ is obtained as

$$C = \begin{pmatrix} \sigma_{11} & \sigma_{12} \\ \sigma_{21} & \sigma_{22} \end{pmatrix},$$

where σ_{11} and σ_{22} represent the variance of CoP-ML$[n]$ and CoP-AP$[n]$, respectively. Similarly, σ_{12} and σ_{21} represent the covariance of CoP-ML$[n]$ and CoP-AP$[n]$. Eigenvalues of the covariance matrix C, denoted by λ_1 and λ_2, are obtained as

$$\lambda_1 = \frac{\sigma_{11} + \sigma_{22} + \sqrt{(\sigma_{11} - \sigma_{22})^2 + 4\sigma_{12}^2}}{2},$$
$$\lambda_2 = \frac{\sigma_{11} + \sigma_{22} - \sqrt{(\sigma_{11} - \sigma_{22})^2 + 4\sigma_{12}^2}}{2}.$$

λ_1 represents the first main component, and λ_2 the second main component. The confidence interval κ is defined as

$$\begin{aligned}\kappa &= \sqrt{-2\ln(1-\alpha)} \\ &= \sqrt{-2\ln 0.05}\end{aligned}$$

where $\alpha = 0.95$ represents the probability of existence of the CoP points within the ellipse. The length of major and minor axes of the confidence ellipse are denoted by $long_{el}$ and $short_{el}$, respectively, which are defined as follows.

$$\begin{aligned}long_{el} &= \kappa\sqrt{\lambda_1} \\ short_{el} &= \kappa\sqrt{\lambda_2}\end{aligned}$$

Using $long_{el}$ and $short_{el}$, log-Area can be obtained as

$$\log\text{-Area} = \log\,(\pi \times long_{el} \times short_{el}).$$

Prieto et al. (1996) utilized the index Area, without taking log-transform, as a sway index[3]. The index Area often used for the comparison between healthy subjects and neurological patients, or between eyes-open and eyes-closed conditions. Rocchi et al. (2002) reported that Area of Parkinson ' s disease (PD) patients with levodopa therapy was larger than healthy subjects, and also than PD patients with deep brain stimulations [4]. On the other hand, Maurer et al. (2003) reported that not only Area of PD patients with levodopa therapy, but also Area of PD patients with deep brain stimulations was also large [5]. Schieppati et al. (1994) reported that Area of PD patients with long duration of illness was larger than healthy subjects [6]. However, Yamamoto et al. (2011) reported that Area of a certain population of PD patients was smaller than healthy subjects [7].

log-Axis1 and log-Axis2 (Indices 4 and 5)

The indices log-Axis1 and log-Axis2 are log of the length of major and minor axis of 95 % confidence ellipse of CoP. These indices are defined as follows.

$$\begin{aligned} \log\text{-Axis1} &= \log long_{el} \\ \log\text{-Axis2} &= \log short_{el} \end{aligned}$$

These indices are rarely used as sway indices, but they include information analogous to log-Area.

Angle (Index 6)

The index Angle represents the absolute value of angle in degree between major axis of 95 % confidence ellipse and the ML-axis. Angle = 0 represents the situation where the major direction of CoP motion is parallel to the ML-axis. Angle = 90 means that the major direction of CoP motion is parallel to the AP-axis. In this way, the index Angle is defined as follows.

$$\text{Angle} = \left| \arctan\left(\frac{\sigma_{12}}{\lambda_2 - \sigma_{11}} \right) \right|$$

Rocchi et al. (2002) reported that the major direction of CoP motion of PD patients with levodopa therapy tended to be parallel to the ML-axis using the index of Angle, and they discussed that an increase in sway in the ML direction might be caused by the decrease in muscle tone of the trunk and hip [4].

Mean-cross-ML and Mean-cross-AP (Indices 7 and 8)

The indices Mean-cross-ML and Mean-cross-AP are the number of events (Mean-crosses) that are defined as the instants of times when CoP-ML and CoP-AP crosses the mean value of CoP-ML_{org} and CoP-AP_{org}, respectively. That is, an mean-cross event occurs when the sign of CoP-ML$[n]$ (CoP-AP$[n]$) and CoP-ML$[n+1]$ (CoP-AP$[n+1]$) change, because the mean of CoP-ML and CoP-AP are set to 0 by the zero-mean operations.

Slope-L-ML and Slope-L-AP (Indices 9 and 12)

The indices Slope-L-ML and Slope-L-AP represent the slopes of log-log plotted power spectral density function (PSD) of CoP-ML and CoP-AP at the low frequency band (about 0.04-0.5 Hz, but this range is determined by a process of linear regression), respectively. These indices characterize the scaling exponents of the power-law shaped PSD at the low frequency band. These indices were calculated using linear regression of PSD at the low frequency band. Details of the regression is described in the next section of the Supplemental Materials. Negatively large values of these indices imply steep power-law behavior in PSD, which means that the low frequency sway predominates the total power. These indices are frequency domain representation of the corresponding slope of stabilogram diffusion plot (SDP) at the long-term regime[8].Yamamoto et al. (2011) reported that a certain population of PD patients exhibited smaller values of Slope-L-AP than healthy subjects [7].

Slope-H-ML and Slope-H-AP (Indices 10 and 13)

The indices Slope-H-ML and Slope-H-AP represent the slopes of log-log plotted power spectral density function (PSD) of CoP-ML and CoP-AP at the high frequency band (about 0.5-3.0 Hz, but this range is determined by a process of linear regression), respectively. These indices characterize the scaling exponents of the power-law shaped PSD at the high frequency band. As in Slope-L-ML/AP indices, these indices were calculated using linear regression of PSD at the low frequency band. As in Slope-L-ML/AP, these indices are frequency domain representation of the corresponding slope of SDP at the short-term regime[8].

Critical-freq-ML and Critical-freq-AP (Indices 11 and 14)

The indices Critical-freq-ML and Critical-freq-AP represent, respectively, the corner frequencies of log-log plotted PSD of CoP-ML and CoP-AP with double-power-law behavior. They are the intersectional frequencies of two regression lines defined above. These indices are frequency domain representation of the corresponding time-lag Critical-Δt-log of SDP.

Zero-cross-V-ML and Zero-cross-V-AP (Indices 15 and 16)

The indices Zero-cross-V-ML and Zero-cross-V-AP are the number of events (Zero-crosses) that are defined as the instants of time when low-pass

filtered CoP-ML or CoP-AP crosses the zeros, respectively, where the cut-off frequency of the filter for CoP velocity was set as 2.5 Hz. Figure S1 exemplifies velocity profiles of CoP-ML and CoP-AP with and without the low-pass-filtering, in which the upper and lower traces represent the velocity profiles of CoP-ML and CoP-AP, respectively. In each trace, gray and black curves represent the velocity profiles with and without the low-pass-filtering, respectively. Vertical red lines represent instants of time when the low-pass-filtered velocity crosses the zero. Zero-cross-V-ML and Zero-cross-V-AP count the number of total zero-cross events in the time-span of 70 seconds.

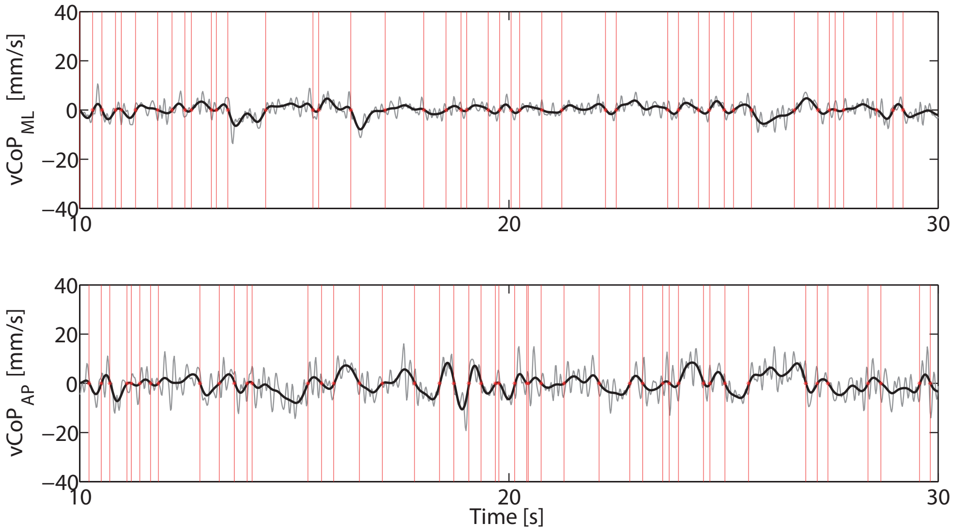

Figure S1: Definition of Zero-cross-V-ML and Zero-cross-V-AP. The upper and lower traces represent the velocity profiles of CoP-ML and CoP-AP, respectively. In each trace, gray and black curves represent the velocity profiles with and without the low-pass-filtering, respectively. Vertical red lines represent instants of time when the low-pass-filtered velocity crosses the zero. Zero-cross-V-ML and Zero-cross-V-AP count the number of total zero-cross events.

log-LNG (Index 17)

The index log-LNG represents the log of the total length of CoP path of planar movement. This index is obtained as follows.

$$\log\text{-LNG} = \log \sum_{n=1}^{N-1} \Big[\big(\text{CoP-ML}[n+1] - \text{CoP-ML}[n]\big)^2 + \big(\text{CoP-AP}[n+1] - \text{CoP-AP}[n]\big)^2\Big]^{1/2}$$

Stylianou et al. (2011) and Chastan et al. (2008) analyzed LNG without taking log-transform as a sway index. Stylianou et al. (2011) reported that LNG of healthy subjects with eyes closed was larger than that with eyes-opened, and LNG of PD patients with eyes-closed was larger than healthy subjects with eyes-closed[9]. Chastan et al. (2008) reported that LNG of both of healthy subjects and PD patients with eyes-closed were larger than that with eyes-opened[10].

log-LNG/Area (Index 18)

The index log-LNG/Area represents the log of the total length of CoP path of planar movement per unit of area. This index is defined as follows by using LNG and Area.

$$\log\text{-LNG/Area} = \log \frac{\text{LNG}}{\text{Area}}$$

Demura et al. (2001) analyzed LNG/Area without taking log-transform as a sway index. They reported that the daily variation of LNG/Area was larger in subjects exhibiting large values LNG/Area than those exhibiting small values[11].

log-Alpha-ML, log-Alpha-AP (Indices 19, 21), Beta-ML and Beta-AP (Indices 20, 22)

The indices log-Alpha-ML (log-Alpha-AP), Beta-ML (Beta-AP) represent the parameters of the probability density function of Gamma distribution, $p(x)$, which is formulated as

$$p(x) = \frac{1}{\Gamma(\alpha)\beta^{\alpha}} x^{\alpha-1} e^{-\frac{x}{\beta}}, \qquad (1)$$

where x represents the inter-zero-cross interval in CoP-ML (CoP-AP) velocity profile, and the parameters β and α provide the index values of Beta-ML

(Beta-AP) and log-Alpha-ML (log-Alpha-AP), respectively. The log-Alpha index is the log transform of α, and Beta index is β itself. To estimate these two parameters, a histogram for sequences of the inter-zero-cross intervals in the CoP-ML (CoP-AP) velocity for a given CoP data was normalized and then fitted by this distribution. Note that the CoP velocity profiles used for the indices of Zero-cross-V-ML and Zero-cross-V-AP (Indices 15 and 16) were low-pass-filtered, whereas those used here were not.

Figure S2 exemplifies a sequence of zero-cross events in a CoP velocity profile for each of CoP-ML and CoP-AP. Histograms and their Gamma-distribution fittings in the figure were prepared using the corresponding inter-zero-cross intervals. With the Gamma-fitted distribution, large and peaky uni-modally distributed small intervals would appear when the CoP velocity profile clearly and predominantly contains the very fast oscillatory components, which can be characterized by small values of Beta combined with large values of log-Alpha. That is, the more frequently and clearly the very fast oscillatory component is contained in the CoP velocity profile, the smaller the values of Beta (β) and the larger the values of log-Alpha (α) are estimated.

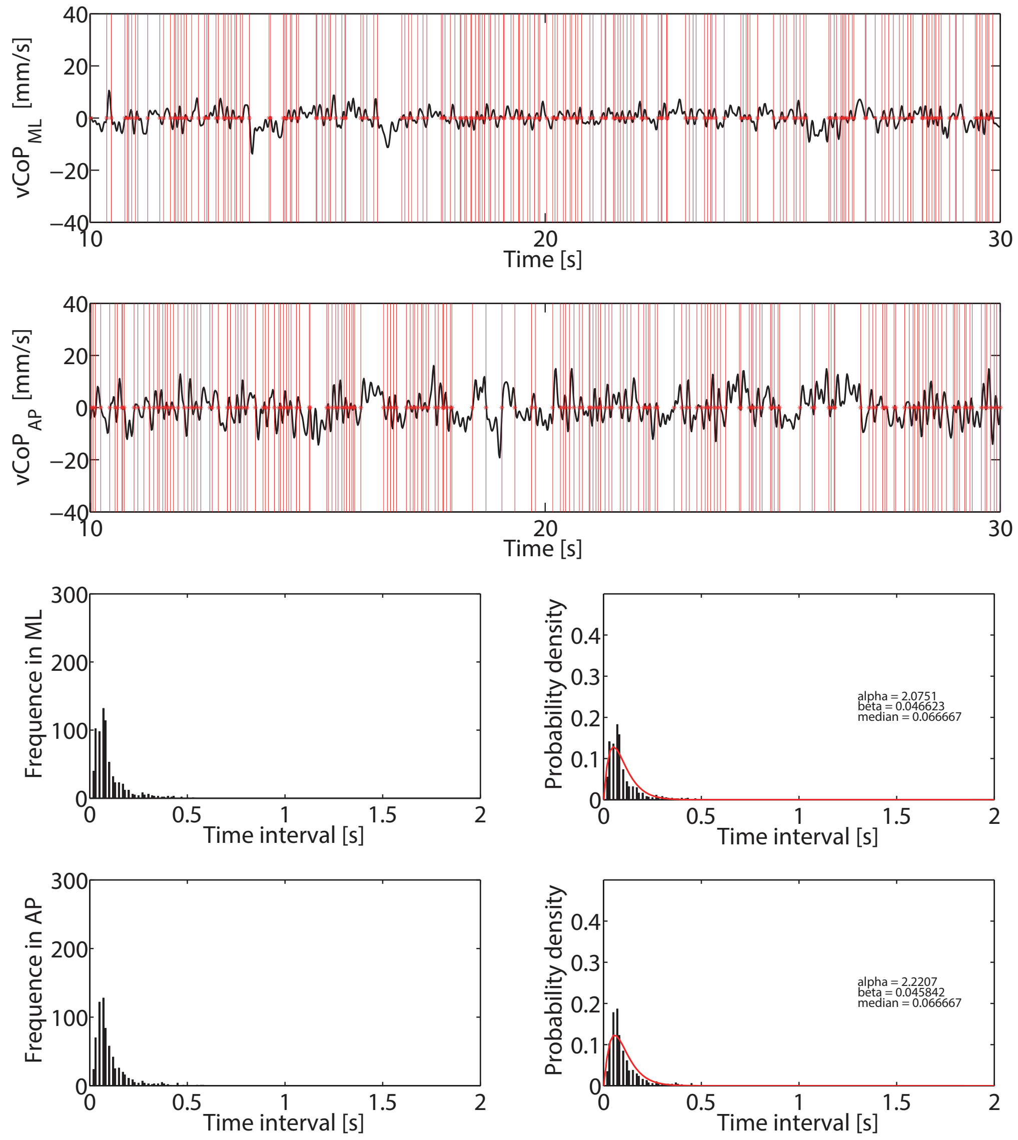

Figure S2: Zero-crosses of CoP velocity and Gamma-distribution fitting of the inter-zero-cross intervals. Upper two traces: CoP-ML and CoP-AP velocity profiles. Vertical red lines represent the occurrences of zero-cross events. Lower left two panels: Histograms of the inter-zero-cross intervals for the CoP-ML and CoP-AP velocity profiles. Lower right two panels: Gamma-distribution fitting as the probability density functions for the histograms.

MT3, Mean-MT (Indices 23, 26), MP3, log-Slope-MP (Indices 24, 27), MD3 and Mean-MD (Indices 25 and 28)

These indices are defined using so called Sway Density Curve (SDC) that characterizes how densely a sway trajectory stays locally in the AP-ML plane as a function of time. More specifically, a SDC represents changes in the time duration of how long CoP trajectory stays locally and time-continuously inside a circle with a radius of R mm, centered at a CoP point at every sampling instant of time[12]. For $R = 3$ mm used for obtaining MT3 and MP3 indices, SDC often exhibited an oscillatory waveform, which means that CoP stays locally for a period of time (corresponding to a peak of the oscillatory SDC waveform), and then migrates to another location (corresponding to a valley of the SDC). See Fig. S3. Since SDC related indices consider peak-values and peak-times of SDC, SDC was low-pass filtered using the fourth-ordered zero-phase-lag Butterworth filter with the cutoff frequency 2.5 Hz. After filtering, the peak detection was conducted. Here, the instance of time for the i-th peak is denoted by $t_p[i]$, and the total number of peaks in the time-span of 70 seconds is denoted by k. The MT3 index represents the mean time interval between successive peaks on SDC with $R = 3$ mm, which is defined as

$$\mathrm{MT} = \frac{1}{k-1}\sum_{i=1}^{k-1}\big(t_p[i+1] - t_p[i]\big).$$

MT indices for other values of the radius R can be obtained similarly.

The index Mean-MT represents the mean value of MT over the radius of circle changing from 2 mm ($R = 2$) to 5 mm ($R = 5$). The index MP3 quantifies the mean of peak values of such oscillatory SDC waveforms for $R = 3$ mm. Denoting a value of the i-th peak of SDC by $peak[i]$, the MP index is defined as

$$\mathrm{MP} = \frac{1}{k}\sum_{i=1}^{k} peak[i]$$

Thus, the larger the values of MP3, the longer time duration CoP trajectory stays locally. MP indices for other values of R can be obtained similarly. The index log-Slope-MP represents the log of the slope of a curve that plots MP values against R from 2 mm to 5 mm. The slope was defined as the slope of regression line of this curve.

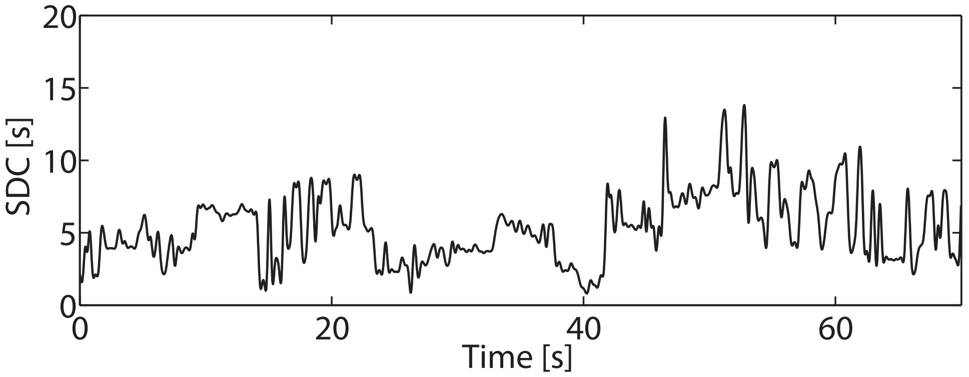

Figure S3: An example of Sway Density Curve (SDC) with $R = 3$ mm.

The index MD3 represents the mean distance in AP-ML plane between two CoP point that correspond to the successive peaks of SDC for $R = 3$. By denoting instances of time of the i-th peak as p, CoP at time p would be $\text{CoP}_{[}p] = (\text{CoP-ML}[p], \text{CoP-AP}[i])$. Then, MD3 index is defined as

$$\text{MD} = \frac{1}{k-1}\sum_{i=1}^{k-1}[\big(\text{CoP-AP}[p+1] - \text{CoP-AP}[p]\big)^2 + \big(\text{CoP-ML}[p+1] - \text{CoP-ML}[p]\big)^2]^{1/2}$$

for $R = 3$ mm. Popa et al. (2007) reported that MD in the patients with chronic lower back pain was larger than that in healthy subjects [13]. The index Mean-MD represents the mean value of MD when the radius of circle R changes from 2 mm to 5 mm.

FD (Index 29)

The index FD represents the fractal dimension of CoP of planar movement. If a CoP path of planar movement is straight line, FD = 1. FD becomes large as CoP moves more randomly. If a CoP movement exhibits a random walk, then FD = 2. For a CoP motion is restricted within a region, FD value would be greater than 2. This index is defined as follows.

$$\text{FD} = \frac{\log N}{\log \frac{Nd}{\text{LNG}}},$$

where d represents the maximum distance between arbitrary two points of CoP on AP-ML plane. Prieto et al. (1996) reported that FD of healthy elderly subjects with eyes-closed was larger than that with eyes-open. Moreover, they also reported that FD in healthy elderly subjects was larger than that in healthy young subjects [3].

log-Area-SW (Index 30)

The index log-Area-SW represents the log of mean of area of triangles formed by two consecutive points of CoP_{org} and the mean position of CoP_{org}. More specifically, this index represents the area enclosed by the CoP path per second, that is, areal velocity of CoP. This index is thus defined as follows.

$$\log\text{-Area-SW} = \log \frac{1}{2T} \sum_{n=1}^{N-1} \big|\text{CoP-ML}[n+1]\text{CoP-AP}[n] - \text{CoP-ML}[n]\text{CoP-AP}[n+1]\big|,$$

where T represents the total time-span (70 s in this study). Prieto et al. (1996) analyzed Area-SW without taking log-transform as a sway index. They reported that Area-SW of elderly healthy subjects was larger than that of young healthy subjects, and Area-SW with eyes-open in both young and elderly was larger than with that with eyes-closed [3]. The areal velocity is usually constant in conservative system, where the principle of conservation of angular momentum is satisfied. That is, the areal velocity is high when the moving radius is small, and it is low when radius is large. However, the phenomenon like this is not observed in CoP movements.

MFREQ, MFREQ-ML and MFREQ-AP (Indices 31, 32 and 33)

The indices MFREQ, MFREQ-ML and MFREQ-AP represent the rotational frequencies of the CoP, and they are defined as follows.

$$\begin{aligned}
\text{MFREQ} &= \frac{\text{MV}}{2\pi \frac{1}{N} \sum_{n=1}^{N} \sqrt{\left(\text{CoP-ML}[n]^2 + \text{CoP-AP}[n]^2\right)}} \\
\text{MFREQ-ML} &= \frac{\text{MV-ML}}{4\sqrt{2} \frac{1}{N} \sum_{n=1}^{N} \big|\text{CoP-ML}[n]\big|} \\
\text{MFREQ-AP} &= \frac{\text{MV-AP}}{4\sqrt{2} \frac{1}{N} \sum_{n=1}^{N} \big|\text{CoP-AP}[n]\big|}
\end{aligned}$$

where MV, MV-ML and MV-AP represent the mean velocities of CoP planar movement, CoP-ML, and CoP-AP, respectively, and they are defined latter of this section. Prieto et al. (1996) reported that both MFREQ and MFREQ-AP in healthy elderly subjects with eyes-closed were larger than those with eyes-open. Moreover, they also reported that both MFREQ and MFREQ-AP in healthy elderly subjects were larger than those in healthy young subjects [3].

log-Power, log-Power-ML and log-Power-AP (Indices 34, 37 and 40)

The indices log-Power, log-Power-ML and log-Power-AP represent the log of total power of CoP of planar movement, CoP-ML, and CoP-AP, respectively. These indices are obtained as the integrated area of PSD from 0.15 Hz to 5 Hz. Denoting the discrete power spectrum density function of CoP of planar movements by $G[m]$, the increment frequency by Δf, the discretized frequency values corresponding to 0.15 Hz and 5 Hz by i and j, respectively, log-Power is defined as follows.

$$\text{log-Power} = \log \sum_{m=i}^{j} m\Delta f G[m]$$

In the same way, log-Power-ML, and log-Power-AP are calculated. Prieto et al. (1996) used Power, Power-ML, and Power-AP without taking log-transform as a sway index, and reported that Power and Power-AP of healthy elderly subjects were larger than those of healthy young subjects, but that Power-ML was independent of age and visual conditions [3].

PF50, PF50-ML and PF50-AP (Indices 35, 38 and 41)

The indices PF50, PF50-ML and PF50-AP represents the median frequencies or the frequencies that bisect the total power of planner CoP, CoP-ML and CoP-AP, respectively. To this end, the smallest discretized frequency value u that satisfies the following equation;

$$\sum_{m=i}^{u} m\Delta f G[m] \geq 0.5\text{Power},$$

is calculated, based on which PF50 is defined as

$$\text{PF50} = u\Delta f,$$

where Δf is the sampling frequency. In the same way, PF50-ML, and PF50-AP are calculated. Prieto et al. (1996) reported that these indices were independent of age and eyes conditions [3].

PF95, PF95-ML and PF95-AP (Indices 36, 39 and 42)

The indices PF95, PF95-ML and PF95-AP repersent the frequencies when 95 % of the total power is found. The smallest discrete value v which satisfied the following equation, is calculated.

$$\sum_{m=i}^{v} m\Delta f G[m] \geq 0.95\text{Power}$$

By using obtained v, PF95 was defined as follows.

$$\text{PF95} = v\Delta f$$

In the same way, PF95-ML and PF95-AP are calculated. Rocchi et al. (2002) reported that PF95 of PD patients without treatment was larger than healthy subjects and PD patients with deep brain stimulation, and that PF95 was correlated with tremor, posture, and gait subcomponents related unified Parkinson's disease rating scale (UPDRS) [4]. Prieto et al. (1996) reported that these indices of healthy elderly subjects with eyes closed were larger than with eyes opened, and that PF95 and PF95-AP of healthy elderly subjects were larger than healthy young subjects regardless of eyes conditions [3].

D-short, D-short-ML and D-short-AP (Indices 43, 47 and 51)

The indices D-short, D-short-ML and D-short-AP represent the diffusion coefficients of CoP of planar movement, CoP-ML, and CoP-AP at short term region, respectively, which can be obtained from SDP on linear-linear scale. These indices are defined as follows.

$$\begin{aligned}\left\langle \Delta\text{CoP}^2 \right\rangle &= 2D_{short}\Delta t \\ \left\langle \Delta\text{CoP-ML}^2 \right\rangle &= 2D_{short\text{-}ML}\Delta t \\ \left\langle \Delta\text{CoP-AP}^2 \right\rangle &= 2D_{short\text{-}AP}\Delta t\end{aligned}$$

These indices are calculated by the slopes at short term regime at $0 \leq \Delta t \leq 1$. If a stochastic process is purely random walk, the diffusion coefficient is 0.5.

$$\begin{aligned}\text{D-short} &= D_{short} \\ \text{D-short-ML} &= D_{short\text{-}ML} \\ \text{D-short-AP} &= D_{short\text{-}AP}\end{aligned}$$

Collins et al. (1993) reported that the open loop control dominating the short term intervals has a higher level of stochastic activity than the closed loop control [14].

D-long, D-long-ML, and D-long-AP (Indices 44, 48 and 52)

The indices D-long, D-long-ML, and D-long-AP represent the diffusion coefficients of CoP of planar movement, CoP-ML, and CoP-AP at long term regime, respectively. As is the case with D-short, these indices are defined as follows.

$$\begin{aligned} \left\langle \Delta \mathrm{CoP}^2 \right\rangle &= 2D_{long}\Delta t \\ \left\langle \Delta \mathrm{CoP\text{-}ML}^2 \right\rangle &= 2D_{long\text{-}ML}\Delta t \\ \left\langle \Delta \mathrm{CoP\text{-}AP}^2 \right\rangle &= 2D_{long\text{-}AP}\Delta t \end{aligned}$$

These indices are calculated by the slopes at long term regime at $2 \leq \Delta t \leq 10$.

$$\begin{aligned} \text{D-long} &= D_{long} \\ \text{D-long-ML} &= D_{long\text{-}ML} \\ \text{D-long-AP} &= D_{long\text{-}AP} \end{aligned}$$

Critical-Δt-linear, Critical-Δt-linear-ML and Critical-Δt-linear-AP (Indices 45, 49 and 53)

The indices Critical-Δt-linear, Critical-Δt-linear-ML and Critical-Δt-linear-AP represent the increment time intervals on the critical point representing a boundary between short term region and long term region of SDP on linear-linear scale of CoP of planar movement, CoP-ML, and CoP-AP, respectively. Collins et al. (1993) reported that these indices reflect time scale of switching between open loop and closed loop control [14]. Maurer et al. (2004) reported that Critical-Δt-linear became small as severity of motor disorder increases [15].

Critical-D-linear, Critical-D-linear-ML and Critical-D-linear-AP (Indices 46, 50 and 54)

The indices Critical-D-linear, Critical-D-linear-ML and Critical-D-linear-AP represent the mean square displacements on the critical point representing a boundary between short term region and long term region of SDP on linear-linear scale of CoP of planar movement, CoP-ML, and CoP-AP, respectively. Collins et al. (1993) reported that these values were relatively

small, and it is associated with a safety limit of CoP that restricts the region of diffusion [14]. Maurer et al. (2004) reported that these indices were not correlated with the severity of motor disorder [15].

Slope-short, Slope-short-ML, and Slope-short-AP (Indices 55, 59 and 63)

The indices Slope-short, Slope-short-ML, and Slope-short-AP represent the slopes (scaling exponents) of Stabilogram Diffusion Plot (SDP) in the log-log scale of CoP of planar movement, CoP-ML, and CoP-AP at short term region, respectively. Mean square values of CoP of planar movement, CoP-ML, and CoP-AP are denoted by $\left\langle \Delta\text{CoP}^2 \right\rangle_{\Delta t}, \left\langle \Delta\text{CoP-ML}^2 \right\rangle_{\Delta t}, \left\langle \Delta\text{CoP-AP}^2 \right\rangle_{\Delta t}$, respectively. Then, these indices are defined using the number of data points m within Δt as follows.

$$\begin{aligned}
\left\langle \Delta\text{CoP}^2 \right\rangle_{\Delta t} &= \frac{1}{N-m} \sum_{n=1}^{N-m} \big(\text{CoP-ML}[n+m] - \text{CoP-ML}[n]\big)^2 + \\
&\quad \big(\text{CoP-AP}[n+m] - \text{CoP-AP}[n]\big)^2 \\
\left\langle \Delta\text{CoP-ML}^2 \right\rangle_{\Delta t} &= \frac{1}{N-m} \sum_{n=1}^{N-m} \big|\text{CoP-ML}[n+m] - \text{CoP-ML}[n]\big|^2 \\
\left\langle \Delta\text{CoP-AP}^2 \right\rangle_{\Delta t} &= \frac{1}{N-m} \sum_{n=1}^{N-m} \big|\text{CoP-AP}[n+m] - \text{CoP-AP}[n]\big|^2
\end{aligned}$$

The value of slope is calculated using a regression line of SDP at short term region. Details of regression is described in the next section of this supplemental material. The slope larger than 1 implies a positively correlated process. That is, increasing or decreasing trends in the past would continue in the future. This type of behavior is known as persistence. On the other hand, the slope smaller than 1 implies negatively correlated process. That is, increasing or decreasing trends in the past would be alternated into opposite trends in the future. This type of behavior is known as anti-persistence. Collins et al. (1993) consider that upright posture is controlled by open loop control at short term region based on the fact that Slope-short is larger than 1 [14].

Slope-long, Slope-long-ML, and Slope-long-AP (Indices 56, 60 and 64)

The indices Slope-long, Slope-long-ML, and Slope-long-AP represent the slopes (scaling exponents) of SDP in the log-log scale of CoP of planar movement, CoP-ML, and CoP-AP at long term region, respectively. As is the case

with Slope-short, details are in the next subsection. Collins et al. (1993) consider that upright postural is controlled by closed loop control at long term region, based on the fact that Slope-long is smaller than 1 [14]. Maurer et al. (2004) reported that the plot of SDP at long term region in PD patients exhibited oscillation with about 1 Hz, and the frequency of this oscillation could be lowered by levodopa therapy or deep brain stimulation [15].

Critical-Δt-log, Critical-Δt-log-ML and Critical-Δt-log-AP (Indices 57, 61 and 65)

The indices Critical-Δt-log, Critical-Δt-log-ML and Critical-Δt-log-AP represent the increment time intervals on the critical point representing a boundary between short term region and long term region of SDP in the log-log scale of CoP of planar movement, CoP-ML, and CoP-AP, respectively. The critical point is calculated as the intersectional time between the two regression lines that fit the plots at two regions. The increment time interval on the critical point for log-log graph are rarely used in previous studies, but those for linear-linear graph are often used [14, 15, 16].

Critical-D-log, Critical-D-log-ML and Critical-D-log-AP (Indices 58, 62 and 66)

The indices Critical-D-log, Critical-D-log-ML and Critical-D-log-AP represent the critical points in the mean square displacements, representing boundaries between short term region and long term region of SDP in the log-log scale of CoP of planar movement, CoP-ML, and CoP-AP, respectively. As in the case with Critical-Δt-log, the critical point for log-log graph are rarely used in previous studies, but those for linear-linear graph are often used [14, 15, 16].

log-RMS, log-RMS-ML, and log-RMS-AP (Indices 67, 68 and 69)

The indices log-RMS, log-RMS-ML, and log-RMS-AP represent the log of the root mean square value of CoP of planar movement, CoP-ML, and

CoP-AP, respectively. These indices are defined as follows.

$$\begin{aligned}
\log\text{-RMS} &= \log\sqrt{\frac{1}{N}\sum_{n=1}^{N}\left(\text{CoP-ML}[n]^2 + \text{CoP-AP}[n]^2\right)} \\
\log\text{-RMS-ML} &= \log\sqrt{\frac{1}{N}\sum_{n=1}^{N}\text{CoP-ML}[n]^2} \\
\log\text{-RMS-AP} &= \log\sqrt{\frac{1}{N}\sum_{n=1}^{N}\text{CoP-AP}[n]^2}
\end{aligned}$$

Rocchi et al. (2002) and Maurer et al. (2004) used RMS, RMS-AP, and RMS-ML without taking log-transform as a sway index. Rocchi et al. (2002) reported that RMS in PD patients with levodopa therapy was larger than healthy subjects and PD patients with deep brain stimulation [4]. Moreover, they also reported that RMS of PD patients without treatment was larger than PD patients with deep brain stimulation. Maurer et al. (2004) reported that RMS in some of PD patients increased by treatments with levodopa therapy, deep brain stimulation, and combination of them than without treatments [15].

log-MV, log-MV-AP, and log-MV-ML (Indices 70, 71 and 72)

The indices log-MV, log-MV-ML, and log-MV-AP represent the log of the mean velocities in CoP of planar movement, CoP-ML, and CoP-AP, respectively. These indices are defined as follows.

$$\begin{aligned}
\log\text{-MV} &= \log\frac{\text{LNG}}{T} \\
\log\text{-MV-ML} &= \log\frac{1}{T}\sum_{n=1}^{N-1}\left|\text{CoP-ML}[n+1] - \text{CoP-ML}[n]\right| \\
\log\text{-MV-AP} &= \log\frac{1}{T}\sum_{n=1}^{N-1}\left|\text{CoP-AP}[n+1] - \text{CoP-AP}[n]\right|
\end{aligned}$$

Prieto et al. (1996) and Kouzaki et al. (2012) used MV, MV-ML, and MV-AP without taking log-transform as a sway index. Kouzaki et al. (2012) reported that MV-AP of healthy elderly subjects was larger than healthy young subjects regardless of eyes condition [17]. Moreover, they reported that MV-AP was positively correlated with physical tremor of soleus.

Flattening (Index 73)

The index Flattening represent the ratio of flattening of 95 % confidence ellipse of CoP. Flattening = 0 implies that the ellipse is equal to a circle. Flattening becomes close to 1 as shape of the ellipse becomes flat. This index is defined as follows.

$$\text{Flattening} = 1 - \frac{short_{el}}{long_{el}}$$

This index is rarely used in previous studies as sway indices.

2. Method for calculating two regression lines with a curve

In this section, the method for fitting a curve (X, Y) in the log-log scale by two regression lines is described for characterizing the double-power-law behavior. In this study, the target band for fitting was 0.125-25 sec in the case of SDP fitting, and 0.04-8 Hz in the case of PSD fitting. These bands in the time and frequency domains correspond to each other. Let us denote the data points in the target band by $\{X_i, Y_i\}$ $i = 1, \cdots, n_{1st} + n_{2nd}$. The target band would be divided into two (small and long term regimes, or low and high frequency bands). Let us denote the number of data points for the first band and the remaining second band for regressions by n_{1st} and n_{2nd}, respectively. Regression line for the 1st band and that for the 2nd band are denoted by L_{1st} and L_{2nd}, respectively. Let us define two regression lines as follows.

$$\begin{aligned} L_{1st} : y &= a_{1st}x + b_{1st}, \\ L_{2nd} : y &= a_{2nd}x + b_{2nd}. \end{aligned}$$

The x coordinate value at the intersection between L_{1st} and L_{2nd} is denoted by x_c. It can be expressed as follows.

$$x_c = \frac{b_{2nd} - b_{1st}}{a_{1st} - a_{2nd}}$$

The total sum of error of regression lines, denoted by e_{total}, is defined as follows.

$$e_{total} = \sum_{i=1}^{n_{1st}} \frac{\{Y_i - (a_{1st}X_i + b_{1st})\}^2}{10^{X_i}} + \sum_{i=n_{1st}+1}^{n_{1st}+n_{2nd}} \frac{\{Y_i - (a_{2nd}X_i + b_{2nd})\}^2}{10^{X_i}}.$$

The constraint condition for calculation of regression lines is $\log_{10} 0.5 \leq x_c \leq \log_{10} 3.0$ in the case of SDP, and $\log_{10} 0.3 \leq x_c \leq \log_{10} 2.0$ in the case of PSD. The coefficients $a_{pre}, b_{pre}, a_{2nd}, b_{2nd}$ were obtained by minimizing e_{total} with the constraint condition.

3. Correlation between MP3 and Zero-cross of CoP velocity

In the result section of the main text, we described the negative correlation between Beta-ML/AP and MP3 indices. A basis of this correlation is actually a positive correlation between MP3 (peak values of SDC) and occurrences of the small zero-cross intervals in CoP velocity profile. Figure S4 exemplifies CoP velocity profiles (ML and AP) with the corresponding SDC waveforms from two subjects. One can confirm from the figure that the occurrence frequency of zero-cross events (either in CoP-ML or CoP-AP velocity profile) tend to be higher when the SDC takes large values (i.e., around peaks of the SDC). High occurrence frequency of zero-cross events implies high occurrence frequency of small zero-cross intervals, from which a histogram for sequences of the inter-zero-cross intervals in the CoP-ML/AP velocity was fitted by PDF of Gamma-distribution. With a Gamma-fitted distribution, large and peaky uni-modally distributed small intervals would appear when the CoP velocity profile clearly and predominantly contains the very fast oscillatory components, which can be characterized by small values of Beta-AP combined with large values of log-Alpha-AP. That is, the more frequently and clearly the very fast oscillatory component is contained in the CoP velocity profile, the smaller the values of Beta-AP (β) and the larger the values of log-Alpha-AP (α) are estimated.

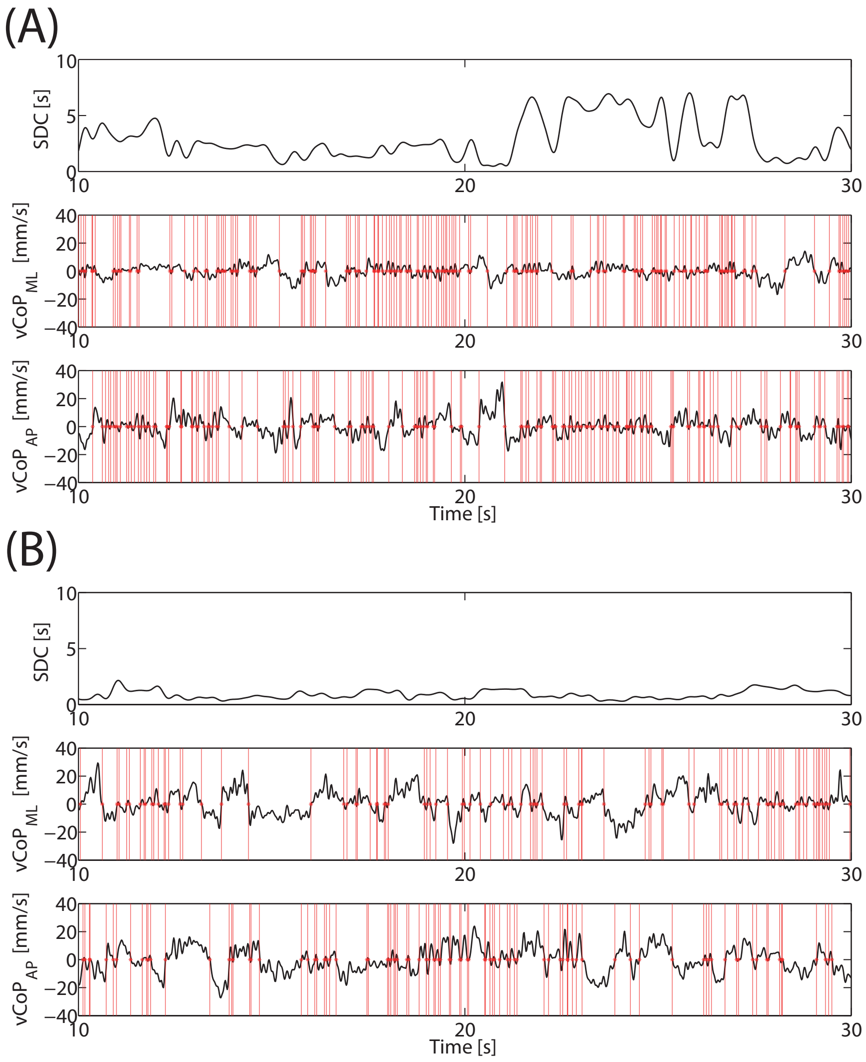

Figure S4: Comparison between SDC and CoP-ML/AP velocity profiles. (A) A sample data from Subject-9. (B) A sample data from Subject-16. In each panel, top trace represents SDC. The second and third traces are the corresponding velocity profiles of CoP-ML and CoP-AP, respectively. Vertical red lines represent the occurrences of zero-cross events.

References

[1] N. Vuillerme, N. Forestier, V. Nougier *et al.*, "Attentional demands and postural sway: the effect of the calf muscles fatigue," *Medicine and science in sports and exercise*, vol. 34, no. 12, pp. 1907–1912, 2002.

[2] R. Kirby, N. Price, and D. MacLeod, "The influence of foot position on standing balance," *Journal of biomechanics*, vol. 20, no. 4, pp. 423–427, 1987.

[3] T. E. Prieto, J. Myklebust, R. Hoffmann, E. Lovett, and B. Myklebust, "Measures of postural steadiness: differences between healthy young and elderly adults," *Biomedical Engineering, IEEE Transactions on*, vol. 43, no. 9, pp. 956–966, 1996.

[4] L. Rocchi, L. Chiari, and F. Horak, "Effects of deep brain stimulation and levodopa on postural sway in parkinson's disease," *Journal of Neurology, Neurosurgery & Psychiatry*, vol. 73, no. 3, pp. 267–274, 2002.

[5] C. Maurer, T. Mergner, J. Xie, M. Faist, P. Pollak, and C. Lücking, "Effect of chronic bilateral subthalamic nucleus (stn) stimulation on postural control in parkinson ' s disease," *Brain*, vol. 126, no. 5, pp. 1146–1163, 2003.

[6] M. Schieppati, M. Hugon, M. Grasso, A. Nardone, and M. Galante, "The limits of equilibrium in young and elderly normal subjects and in parkinsonians," *Electroencephalography and Clinical Neurophysiology/Evoked Potentials Section*, vol. 93, no. 4, pp. 286–298, 1994.

[7] T. Yamamoto, Y. Suzuki, K. Nomura, T. Nomura, T. Tanahashi, K. Fukada, T. Endo, and S. Sakoda, "A classification of postural sway patterns during upright stance in healthy adults and patients with parkinson's disease." *JACIII*, vol. 15, no. 8, pp. 997–1010, 2011.

[8] H. van der Kooij, E. van Asseldonk, and F. C. van der Helm, "Comparison of different methods to identify and quantify balance control," *Journal of neuroscience methods*, vol. 145, no. 1, pp. 175–203, 2005.

[9] A. P. Stylianou, M. A. McVey, K. E. Lyons, R. Pahwa, and C. W. Luchies, "Postural sway in patients with mild to moderate parkinson's

disease," *International Journal of Neuroscience*, vol. 121, no. 11, pp. 614–621, 2011.

[10] N. Chastan, B. Debono, D. Maltête, and J. Weber, "Discordance between measured postural instability and absence of clinical symptoms in parkinson's disease patients in the early stages of the disease," *Movement Disorders*, vol. 23, no. 3, pp. 366–372, 2008.

[11] S. Demura, S. Yamaji, M. Noda, T. Kitabayashi, and Y. Nagasawa, "Examination of parameters evaluating the center of foot pressure in static standing posture from the viewpoints of trial-to-trial reliability and interrelationships among parameters," *Equilibrium Research*, vol. 60, no. 1, pp. 44–55, 2001.

[12] M. Jacono, M. Casadio, P. G. Morasso, and V. Sanguineti, "The sway-density curve and the underlying postural stabilization process," *MOTOR CONTROL-CHAMPAIGN-*, vol. 8, pp. 292–311, 2004.

[13] T. Popa, M. Bonifazi, R. Della Volpe, A. Rossi, and R. Mazzocchio, "Adaptive changes in postural strategy selection in chronic low back pain," *Experimental brain research*, vol. 177, no. 3, pp. 411–418, 2007.

[14] J. J. Collins and C. J. De Luca, "Open-loop and closed-loop control of posture: a random-walk analysis of center-of-pressure trajectories," *Experimental Brain Research*, vol. 95, no. 2, pp. 308–318, 1993.

[15] C. Maurer, T. Mergner, and R. Peterka, "Abnormal resonance behavior of the postural control loop in parkinson's disease," *Experimental brain research*, vol. 157, no. 3, pp. 369–376, 2004.

[16] C. Maurer and R. J. Peterka, "A new interpretation of spontaneous sway measures based on a simple model of human postural control," *Journal of Neurophysiology*, vol. 93, no. 1, pp. 189–200, 2005.

[17] M. Kouzaki and K. Masani, "Postural sway during quiet standing is related to physiological tremor and muscle volume in young and elderly adults," *Gait & posture*, vol. 35, no. 1, pp. 11–17, 2012.
